# A Novel Interaction Network Used by Potyviruses in Virus–Host Interactions at the Protein Level

**DOI:** 10.3390/v11121158

**Published:** 2019-12-14

**Authors:** Marjo Ala-Poikela, Minna-Liisa Rajamäki, Jari P.T. Valkonen

**Affiliations:** Department of Agricultural Sciences, University of Helsinki, P.O. Box 27, FI-00014 Helsinki, Finland; alapoikela@gmail.com

**Keywords:** potyvirus, potato virus A, VPg, HCpro, eIF4E, eIF(iso)4E, interaction network

## Abstract

Host proteins that are central to infection of potyviruses (genus *Potyvirus*; family Potyviridae) include the eukaryotic translation initiation factors eIF4E and eIF(iso)4E. The potyviral genome-linked protein (VPg) and the helper component proteinase (HCpro) interact with each other and with eIF4E and eIF(iso)4E and proteins are involved in the same functions during viral infection. VPg interacts with eIF4E/eIF(iso)4E via the 7-methylguanosine cap-binding region, whereas HCpro interacts with eIF4E/eIF(iso)4E via the 4E-binding motif YXXXXLΦ, similar to the motif in eIF4G. In this study, HCpro and VPg were found to interact in the nucleus, nucleolus, and cytoplasm in cells infected with the potyvirus potato virus A (PVA). In the cytoplasm, interactions between HCpro and VPg occurred in punctate bodies not associated with viral replication vesicles. In addition to HCpro, the 4E-binding motif was recognized in VPg of PVA. Mutations in the 4E-binding motif of VPg from PVA weakened interactions with eIF4E and heavily reduced PVA virulence. Furthermore, mutations in the 4G-binding domain of eIF4E reduced interactions with VPg and abolished interactions with HCpro. Thus, HCpro and VPg can both interact with eIF4E using the 4E-binding motif. Our results suggest a novel interaction network used by potyviruses to interact with host plants via translation initiation factors.

## 1. Introduction

The genus *Potyvirus* (family Potyviridae) contains the largest number of plant-infecting RNA viruses that cause yield losses in all major crops [1]. The potyvirus genome is a monopartite, positive-strand RNA—(+)ssRNA—that contains the viral genome-linked protein (VPg) covalently bound to its 5’ end [2,3]. The genome contains a single open reading frame encoding a polyprotein that is proteolytically cleaved into 10 mature proteins by three virus-encoded proteinases: nuclear inclusion protein a (NIa), helper component-proteinase (HCpro), and protein 1 (P1) [1]. One or two additional proteins are produced by a frame-shifting mechanism [4,5,6,7].

The most comprehensively studied recessive genes used to breed resistance to potyviruses in crop plants encode variants of eukaryotic translation initiation factor 4E (eIF4E) or iso4E [eIF(iso)4E] [8,9,10]. In this paper, we refer to both translation initiation factors with the abbreviation 4E, unless the factor needs to be specified.

In the many virus-host combinations in which the potyvirus is able to overcome 4E-mediated resistance, this is caused by mutations in the central part of VPg. One hypothesis suggests that VPg imitates the cap to support viral translation, and/or it inhibits host translation by sequestering 4E [8,9,10,11], the limiting factor for translation [12]. Consequently, the absence of an interaction between VPg and 4E would render the virus unable to complete its infection cycle. Indeed, mutations in 4Es that confer resistance to potyviruses often alter the VPg–4E interaction [13], such that the cap-binding function remains but binding of VPg is no longer possible [14,15,16].

The scaffold protein eIF4G contains a specific motif for 4E binding with a consensus sequence YXXXXLΦ, where X is a variable amino acid and Φ is a hydrophobic residue [17]. In addition to this canonical motif, recent data indicate that 4G interacts with 4E via a second, non-canonical motif at the lateral surface of 4E that strengthens the interaction [18]. eIF4G binds to eIF4E and eIF(iso)4G binds to eIF(iso)4E to form the host eIF4F and eIF(iso)4F complexes, respectively, which also include the ATP-dependent RNA helicase eIF4A. The eIF4F-mRNA complex engages the 40S small ribosomal subunit bound to the methionine initiator transfer RNA (tRNA) and additional translation factors to yield the translation pre-initiation complex [19,20]. In addition to eIF4G, other 4E-binding proteins involved in translation regulation and nuclear transport bind to 4E through the YXXXXLΦ motif in mammals [17], thus preventing binding of 4E to eIF4G and the initiation of cap-dependent translation [17,21]. Plants lack 4E-binding protein homologs; however, a plant-specific protein, conserved binding of eIF4E 1 (CBE1), which contains a 4E-binding motif, was recently identified. CBE1 is a constituent of 4E cap-binding complexes and has the potential to regulate gene expression [22]. Plant 4Es can also be phosphorylated at the lateral surface of 4E by the energy-sensing kinase SnRK1, which inhibits translation [23]. Phosphorylation of 4E increases binding of VPg [24].

HCpro is another potyvirus protein that interacts with 4Es [25]. HCpro contains the canonical 4E-binding motif found in eIF4G, and mutations in this motif in HCpro of PVA abolish viral infectivity [25]. HCpro is involved in viral genome amplification [26], RNA binding [27], viral cell-to-cell and long-distance movement [28], and suppression of the basal antiviral defense mechanism based on RNA interference (RNAi) [29,30,31], and RNA decay [32]. VPg is also involved in these functions during the viral infection cycle [2,27,32,33,34,35,36]. Furthermore, both VPg and HCpro are multifunctional potyviral proteins that form homodimers [3,37] and interact with each other [38,39].

Although interactions between VPg and 4E during translation initiation and interactions between HCpro and 4E in association with replication/translation vesicles induced by the viral protein 6K2 contribute to potyvirus infection, such interactions alone cannot completely explain the role of these host factors in virus infection. First, the role of the VPg–4E interaction in potyvirus translation is obscured because the 5’ untranslated region of the potyvirus RNA can promote translation of the viral polyprotein in a cap-independent manner by directly recruiting ribosomal subunits, which is, thus, independent of eIF4E [40,41]. Second, 4E-mediated resistance can be associated with defects in cell-to-cell movement of the virus, as found in pepper and pea plants that show recessive resistance to potato virus Y and pea seed-borne virus, respectively, owing to mutations in *eIF4E* genes [14,42]. 4E-mediated resistance to lettuce mosaic virus conferred by *mo1* in lettuce is related to defective long-distance movement of this virus [43]. In these cases, it is not obvious that a connection exists between the role of 4E in translation initiation and the mechanism by which 4E confers resistance. Third, mutations in protein 1 (P1) [44], protein 3 (P3) [45], or the cylindrical inclusion protein [46,47] of potyviruses also can break eIF4E-mediated resistance in certain virus–host combinations. Furthermore, eIF4E has also been detected in the nucleus of plant and animal cells, suggesting its role in other processes [21,48].

HCpro and VPg interact with 4E and each other. Both proteins are involved in the same functions during viral infection and therefore, we considered that the proteins might have coordinated functions during potyviral infection. We therefore characterized interactions of HCpro and VPg with each other and with 4Es in plants. Our results indicated that HCpro and VPg interact with each other in the nucleus, nucleolus, and cytoplasm. Similar to HCpro, the VPg of PVA and of some other potyviruses contains the canonical 4E-binding motif, and mutations in this motif of PVA VPg affected interactions with 4E and heavily reduced PVA infectivity. Hence, HCpro, as well as VPg, can bind 4E using its 4E-binding motif and, additionally, VPg binds 4E via the cap-binding pocket. Our results also suggest that interactions of VPg and HCpro with 4E dominate the mutual interaction of VPg and HCpro. In addition, our results imply that 4E can form tertiary complexes with the aforementioned potyvirus proteins. Taken together, these results suggest a novel interaction network by which a virus interacts with components of the host translation system.

## 2. Materials and Methods

### 2.1. Yeast Hybrid Systems

Standard molecular biology protocols were used unless specified [49]. The full-length coding region of VPg and its truncated versions were amplified from the full-length infectious cDNA clone PVA-B11 (AJ296311) [50] with primers that introduced restriction sites (Table S1). PCR products were cloned into yeast two-hybrid (Y2H) vectors pGADT7 or pGBKT7 for fusion with the activation domain (AD) or binding domain (BD), respectively (Clontech, Mountain View, CA, USA). Cloning of eIF4Es and eIF(iso)4Es from tobacco and potato and of PVA HCpro and its truncated forms for Y2H assays has been described [25]. The sequences of the tobacco (T) and potato (P) 4Es have the following accession numbers: T4Ea (AY702653), T4Eb (FN666433), Tiso4Ea (FN666434), Tiso4Eb (AY699609), P4Ea (FN666435), P4Eb (FN666436), Piso4Ea (FN666437), and Piso4Eb (FN666438) in the EMBL sequence database. All clones and inserts in plasmids were verified by restriction analysis and sequencing.

The Matchmaker GAL4 Two-Hybrid System, including supplier-provided positive and negative controls, was used for the Y2H assays following the Clontech Yeast Protocols Handbook (Clontech). Interaction between GAL4 fusion proteins was evaluated via growth assays for *Saccharomyces cerevisiae* strain AH109 [25] and *S. cerevisiae* strain PJ69-4A [51] as described. A small-scale lithium acetate yeast procedure (Clontech) was used to co-transform yeast cells simultaneously with the BD and AD fusion vectors. Culture medium lacking leucine and tryptophan was used to select for positive transformants, and protein interactions were observed on plates with growth medium lacking adenine, histidine, leucine, and tryptophan. Yeast growth at 30 °C was observed for up to 14 days.

For yeast three-hybrid (Y3H) assays, the coding sequences for full-length VPg, HCpro, and translation initiation factors were amplified from the Y2H vectors using primers that included the sites for *Nru*I and *Sal*I (Table S1). The PCR products were cloned into the Y3H expression vector pRED-NLSa (courtesy of P.B.F. Ouwerkerk, Leiden University, Leiden, The Netherlands) [52]. *S. cerevisiae* cells (strain PJ69-4A) [51] were co-transformed with BD, AD, and pRED-NLSa expression vectors, as above. Synthetic minimal medium lacking leucine, tryptophan, and uracil was used to select for the input plasmids. Positive transformants were selected for protein interactions by growth on medium lacking uracil, histidine, leucine, and tryptophan in the absence and presence of 1 mM 3-amino-1,2,4-triazole (3-AT). Yeast cultures were grown to OD_600_ = 1 and diluted 10- and 100-fold before being spread (2 μL) on selection plates. Yeast growth at 30 °C was observed for up to 10 days.

### 2.2. Bimolecular Fluorescence Complementation (BiFC) Assay and Fluorescently Tagged Proteins

The VPg double-alanine substitution mutant Y89A;L94A was produced using the Quick-Change Site-Directed Mutagenesis kit (Agilent Technologies, Foster City, CA, USA) using a 1898-nucleotide-long *Hin*dIII-*Apa*I fragment of PVA-B11 cDNA that included the VPg-encoding sequence in the vector pBluescript [34]. The resulting construct was designated as pBLUe-VPgY89A;L94A. Mutations were introduced into the VPg sequence with primers (Table S2) as recommended by Agilent. The wild-type and mutated VPg sequences were amplified using primers (Table S2) from pBLUE vectors and cloned into the pRT-YN and pRT-YC expression cassettes, which include the 35S promoter [53] to express the N-proximal (YFP_1–154_) and C-proximal (YFP_155–239_) parts of yellow fluorescent protein (YFP), respectively, fused to the N and C terminus of VPg and its mutants. Cloning of eIF4E and eIF(iso)4E of tobacco and HCpro for BiFC assays has been described [25]. Mutations in T4Ea resulting in the double amino acid substitutions E83D;W86R and W115A;E116A were introduced directly into T4Ea in vectors pRT-YN and pRT-YC using the QuikChange Site-Directed Mutagenesis kit and primers for introducing mutations (Table S2).

HCproY345A;L350A was amplified from PVA-HCE4mut [25] using primers (Table S2) and subcloned into pRT-YN. All pRT vectors were digested with *Hin*dIII to release the expression cassette including the 35S promoter and subcloned into the binary vector pLH7000 that had been previously linearized with *Hin*dIII for agroinfiltration and BiFC [53].

To construct the HCpro red fluorescent protein (RFP) fusion pLH-RFP-HCpro, the PVA HCpro coding sequence was amplified from an infectious cDNA clone of PVA-B11 with primers (Table S2) that introduced *Nco*I sites and simultaneously ligated into monomeric red fluorescent protein (mRFP) containing *Xho*I and *Pci*I sites, as described [54]. Mutations Y345A;L350A were introduced into pA-HCpro [55] using primers as described [25], and the resulting plasmid was designated pAHCproY345A;L350A.

The PVA VPg coding sequence was amplified from an infectious cDNA clone of PVA-B11, and the mutated VPg was amplified from pBLUe-VPgY89A;L94A and subcloned into the binary vector pLH-RFP [54] using the *Xho*I site and primers (Table S2). The constructs were designated pLH-VPg-RFP and pLH-VPgY89A;L94A-RFP, respectively [25]. To obtain pA-VPg, the HC-Pro fragment in pA-HCpro was replaced by the aforementioned PCR product. Mutations Y89A;L94A were introduced directly with primers as described above (Table S2) to construct pA-VPgY89A;L94A.

To make the green fluorescent protein (GFP) construct pA-Tiso4E-GFP, the full-length Tiso4Eb (AY699609) coding sequence was amplified from the Y2H vector with primers that introduced *Xho*I and *Nco*I restriction sites (Table S2), and this fragment was then cloned into pRT-VPg-GUS-GFP to replace VPg-β-glucuronidase (VPg-GUS) [36]. The expression cassette including the 35S promoter was subsequently subcloned using *Hin*dIII sites into the binary vector pKOH200.

To construct PVAVPgY89A;L94A-GFP, the 1898-nucleotide-long *Hin*dIII-*Apa*I fragment of the aforementioned pBLUe-VPgY89A;L94A was cloned into pUC35SPVAgfp using the unique *Apa*I and *Swa*I restriction sites [34] to substitute wild-type VPg for VPgY89A;L94A. Subsequently the resulting PVA cDNA was transferred into the binary vector pCAMBIA0390 using the *Kpn*I and *Sal*I sites as described previously [25].

The infectious cDNA clone of PVA-B11 expressing YN-fused HCpro was constructed as described [25]. 6K2-RFP was introduced between NIa and CP of the aforementioned cDNA clone of PVA-B11 expressing YN-HCpro, and the new infectious clone was designated PVA-ynHC/6Krfp [54]. All clones were verified by restriction enzyme analysis and sequencing. *Arabidopsis thaliana* Fib2-RFP was provided courtesy of M. Taliansky, The James Hutton Institute, Scotland.

### 2.3. Virus Inoculation and Agroinfiltration

The PVA cDNA clones were inoculated biolistically into two full-grown leaves of *Nicotiana benthamiana* Domin and *Nicotiana tabacum* L. (cv. Samsun nn) using HandyGun, as described [56]. Gold particles (diameter, 1.0 µm; Bio-Rad, Hercules, CA, USA) were coated with the PVA plasmid, with 0.5 μg plasmid DNA used for each bombardment. Plants were grown in a growth chamber under controlled conditions (Weiss Umweltstechnik; photoperiod, 16 h; light intensity, 250 µE s^−1^ m^−2^; temperature, 22/18 °C day/night; relative humidity, 75%), watered every second day, and fertilized weekly with a 1% (N/P/K = 16:9:22.5) fertilizer (Yara, Espoo, Finland).

The presence of PVA was assessed in the upper non-inoculated leaves by double-antibody sandwich enzyme-linked immunosorbent assay (DAS-ELISA) using a PVA-specific monoclonal antibody (58/0; SASA, Edinburgh, UK) as described [57]. Leaf samples were weighed and ground in ELISA sample buffer at 1 g/10 mL, and aliquots of 100 µL were transferred to a microtiter plate coated with 58/0. Known amounts of purified PVA virions were included for comparison to estimate virus concentrations.

For agroinfiltration, binary vectors were transformed into *Agrobacterium tumefaciens* (pGV2260) by electroporation. The OD_600_ was adjusted to 0.5 with infiltration medium [25] and the cultures of the two *A. tumefaciens* strains carrying BiFC constructs were combined in equal volumes for infiltration. Likewise, individual proteins with fused fluorescent tags were diluted to OD_600_ = 0.5 before infiltration, and the infectious full-length clones were diluted to OD_600_ = 0.3.

### 2.4. Microscopy

YFP, GFP, and RFP fluorescence was observed with an Axio Imager.M2 microscope (Carl Zeiss Microscopy GmbH, Jena, Germany) with an epifluorescence HXP 120 illuminator (Zeiss) at 2–3 days post-infiltration (dpif). A GFP filter (Zeiss) with bandpass 470/40 nm for excitation and 525/50 nm for emission was used for GFP and YFP, and an RFP filter with bandpass 546/12 nm for excitation and 575–640 nm for emission was used for RFP. Images were acquired with an AxioCamMR3 controlled by ZEN 2012 blue edition software (Zeiss).

Confocal microscopy was carried out 3 dpif with a Leica TCS SP2 AOBS device using a 63× water immersion objective at the Institute of Biotechnology, University of Helsinki, as described [25], and the resulting images were analyzed using Leica LAS AF Lite.

### 2.5. Western Blot Analysis

Proteins from agroinfiltrated *N. benthamiana* leaves and co-transformed yeast cells were extracted as described [25]. Proteins in whole-cell lysates were subjected to sodium dodecyl sulfate (SDS) -polyacrylamide gel electrophoresis (12% or 15% polyacrylamide gels) and transferred to a Hybond-P nitrocellulose membrane (GE Healthcare, Buckinghamshire, UK) by electroblotting. Polyclonal anti-HCpro (courtesy of F. Rabenstein, Julius Kühn-Institut, Quedlinburg, Germany) or polyclonal anti-eIF4E (courtesy of K. Browning, University of Texas, Austin, TX, USA) was used for plant samples, and a monoclonal antibody against the GAL4 AD or GAL4 DNA BD (1:50,000; Clontech, Mountain View, CA, USA) was used for yeast samples. Bound polyclonal antibodies were detected with horseradish peroxidase–conjugated anti-rabbit serum (1:250,000; GE Healthcare, Buckinghamshire, UK), and monoclonal antibodies were detected with horseradish peroxidase-conjugated anti-mouse serum (1:200,000; GE Healthcare). Detection was carried out with the Super Signal West Femto Chemiluminescent Substrate for detection of horseradish peroxidase (Thermo Scientific, Rockford, IL, USA) and visualized by exposure to X-ray film. Equal loading was controlled by Coomassie staining.

### 2.6. RNA Silencing Suppression Assay

Leaves of the transgenic line 16c of *N. benthamiana* [30] were co-infiltrated “on the spot” [58] with *A. tumefaciens* (pGV2260) transformed for expression of different proteins. For infiltration, *A. tumefaciens* cultures for expression of mgfp4 [36], HCpro, HCproY345A;L351A, VPgY89A;L94A, and Tiso4E were diluted with infiltration medium to a final OD_600_ = 0.5 and for co-infiltration, the cultures were combined at equal ratios. If any of these constructs were not needed for a given experiment, the corresponding *A. tumefaciens* strain was replaced with a strain expressing GUS [55]. GFP accumulation in the infiltrated leaves was detected using a hand-held UV-lamp (B-100 AP; UVP). Images were taken with an EOS 40D digital camera (Canon, Amsterdam, The Netherlands) and processed using Corel PHOTO-PAINT X5 (Corel Corporation, Ottawa, Canada).

### 2.7. Northern Blot Analysis

Relative amounts of high-molecular-weight (HMW) and low-molecular-weight (LMW) RNA were assayed in the agroinfiltrated leaf areas by northern analysis. Total RNA was isolated from frozen leaf material using Trizol (Invitrogen, Carlsbad, CA, USA). HMW and LMW RNAs were separated into different fractions using LiCl precipitation, and an antisense RNA probe for detection of *gfp* mRNA and siRNA was prepared and radioactively labeled with [α-^32^P]UTP (PerkinElmer) [55]. LMW RNA (1.5–2 µg) was separated using denaturing urea polyacrylamide gel (15%) electrophoresis, electroblotted onto Hybond-NX nylon membrane (GE Healthcare), and crosslinked using 1-ethyl-3-(3-dimethylaminopropyl) carbodiimide [59]. HMW RNA (3–5 µg) was analyzed by agarose-formaldehyde gel electrophoresis, transferred onto Hybond-NX nylon membranes (GE Healthcare) by capillary blotting, and crosslinked with UV light [49]. Hybridization, washing, and autoradiography were done as described [55]. Experiments were repeated two times.

Radioactive signals were detected using the IP screen (Kodak) and PhosphorImager (FLA-5001, Fuji, Tokyo, Japan). Relative intensities (signals) were quantified using Bio-Rad Quantity One v4.6.9 software (www.Bio-Rad.com). Signals were normalized to RNA loading amount.

### 2.8. Multiple Sequence Alignment and Protein Structure Prediction

Amino acid sequences of VPg retrieved from the DPVWeb database (http://www.dpvweb.net/) [60], and ClustalW2 and Jalview were used for visualization and editing of the aligned sequences [61]. Three-dimensional structures of eIF4E and its mutants were predicted using I-TASSER [62] as described and analyzed using PyMOL (The PyMOL Molecular Graphics System, Version 1.5.0.4, Schrödinger, LLC).

## 3. Results

### 3.1. VPg and HCpro Interact in the Nucleus and Nucleolus during Viral Infection

**Figure 1:**
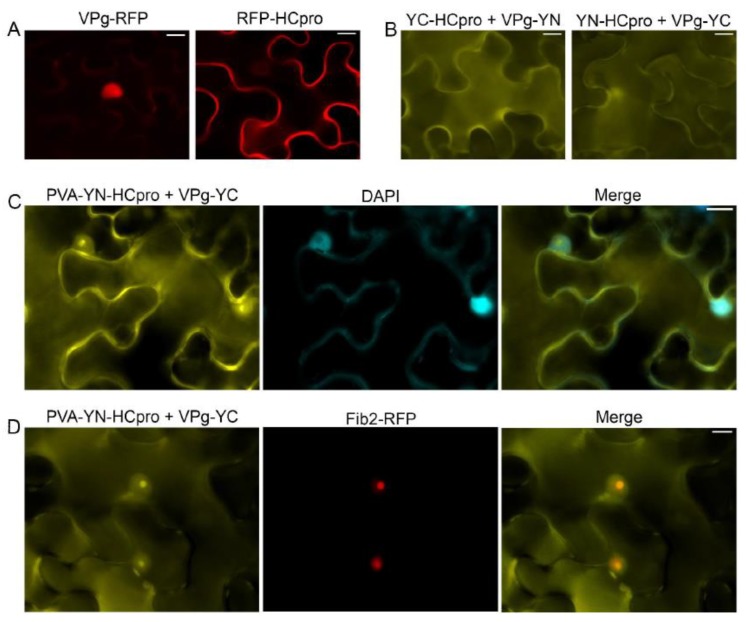
Subcellular localization of potyviral genome-linked protein (VPg) and helper component proteinase (HCpro) and their interaction as observed in epidermal cells of *Nicotiana benthamiana* by epifluorescence microscopy. (**A**) Potato virus A (PVA) VPg fused to monomeric red fluorescent protein (mRFP) is detected in the nucleus, and HCpro fused to mRFP is detected in the cytoplasm. (**B**) VPg and HCpro were separately fused to the C-proximal (YC) and the N-proximal (YN) parts of the YFP. The interaction of VPg with HCpro in the cytoplasm and nucleus was assessed with BiFC. Reciprocal combinations of YC- and YN-fused proteins were analyzed. (**C**) The interaction of VPg with HCpro and its co-localization with DAPI-stained nuclei. YN-HCpro was expressed from the engineered infectious clone PVA, and YC-VPg was co-expressed from a binary pLH vector under the 35S promoter. (**D**) The interaction of VPg with HCpro and its co-localization with the nuclear protein fibrillarin fused to RFP. YN-HCpro was expressed from the engineered infectious clone PVA, and YC-VPg was co-expressed from a binary pLH vector under the 35S promoter. BiFC was observed 3 days post-infiltration (dpif). Scale bars represent 10 µm.

Expression of VPg-RFP in the leaves of *N. benthamiana* after agroinfiltration revealed red fluorescence predominantly in the nucleus, whereas RFP-HCpro expressed in the same manner was detected mainly in the cytoplasm (Figure 1A). The VPg–HCpro interaction was detected in the cytoplasm and in a subcellular compartment that was presumably the nucleus by epifluorescence microscopy (Figure 1B) using the BiFC assay [53] with HCpro and VPg tagged with the C- or N-proximal half of the YFP. Subsequently, YN-HCpro was expressed from an engineered infectious PVA clone placed under the cauliflower mosaic virus 35S promoter (PVA-YN-HCpro) [25]. PVA-YN-HCpro and VPg-YC were co-expressed in full-grown leaves of *N. benthamiana*, and interaction of HCpro and VPg was detected in the cytoplasm, nucleus, and nucleolus, as verified by staining nuclei with 4’,6-diamidino-2-phenylindole (DAPI) (Figure 1C). When PVA-YN-HCpro, VPg-YC, and Fib2-RFP were co-expressed, the signals for YFP and RFP co-localized in the nucleus and nucleolus, further confirming localization of HCpro and VPg in the nucleus and nucleolus (Figure 1D).

**Figure 2:**
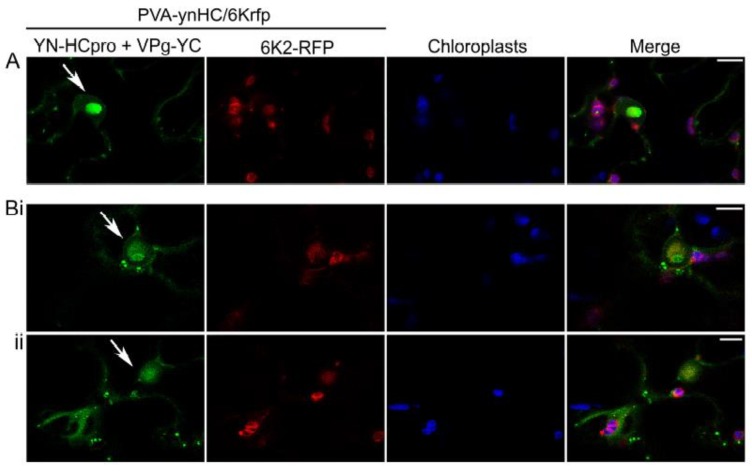
Detection of subcellular localization of the interaction between VPg and HCpro using confocal microscopy. YN-HCpro was expressed from the engineered infectious clone PVA-ynHC/6Krfp, and VPg-YC was co-expressed from a binary pLH vector under the 35S promoter. The 6K2 protein fused to RFP was also expressed from PVA-ynHC/6Krfp. (**A**,**B**) Confocal images captured at 3 dpif represent high-magnification of single optical sections from separate z-series. Separate single optical sections from the same z-series are indicated by **i** and **ii**. Arrows indicate the nucleus. Scale bars represent 10 µm.

Another engineered, infectious PVA clone placed under the 35S promoter (PVA-ynHC/6Krfp) was agroinoculated into leaves of *N. benthamiana* to co-express HCpro fused with YN (YN-HCpro) and the viral protein 6K2 tagged with RFP (6K2-RFP; expressed from the NIb/CP junction of the viral polyprotein) [54]. Signals of interaction between YN-HCpro and YC-VPg (shown in green) were observed by confocal microscopy in punctate cytoplasmic bodies in PVA-infected cells and in the periphery of the nucleus and chloroplasts but were rarely associated with RFP fluorescence expressed in the 6K2-RFP-induced replication vesicles associated with chloroplasts (Figure 2).

### 3.2. Protein Regions Involved in the Interaction between VPg and HCpro

**Figure 3:**
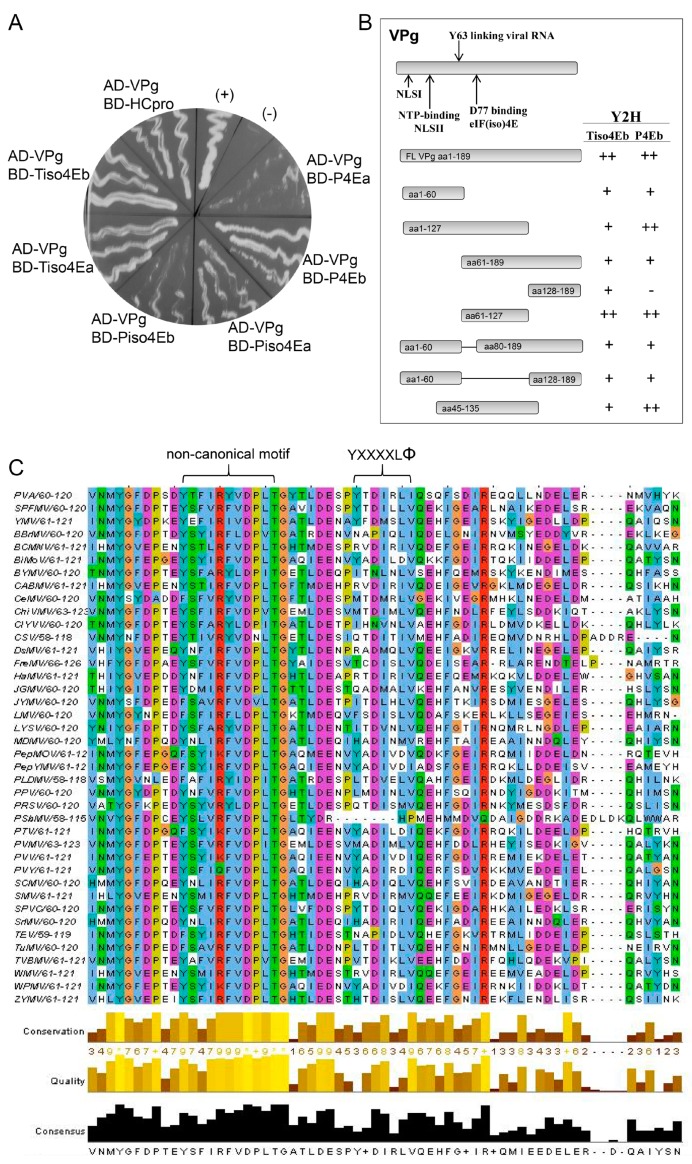
PVA VPg contains a putative eIF4E-binding motif. (**A**) Yeast growth (*Saccharomyces* *cerevisiae*, strain AH109) 14 days after plating on selective medium supplemented with 1 mM 3-AT (Petri dish, 8.5 cm) indicates an interaction between the tested proteins. VPg was tested for interactions with different forms of translation initiation factors eIF(iso)4E (iso4E) and eIF4E (4E) from both tobacco (T) and potato (P): Tiso4Ea and b, Piso4Ea and b, and P4Ea and b [25]. Possible interactions were detected when VPg was fused to the AD and the translation initiation factors were fused to the BD. No interactions were detected when VPg was fused to the BD and the translation initiation factors were fused to the AD. The interaction of VPg with HCpro of PVA was used as an additional positive control and was consistent with the previous results obtained with the LexA Y2H [63]. (+) and (−) are supplier-provided positive and negative controls, respectively. (**B**) Deletion mapping of the regions in VPg involved in the interaction with translation initiation factors using Y2H. The amino acids in the full-length (FL) and truncated forms of VPg are shown. Interaction results are a summary of tests in both the AD and BD vectors and are shown to the right: ++, efficient yeast growth similar to the GAL4 positive control; +, moderately efficient yeast growth; −, no detectable growth. (**C**) Multiple sequence alignment of the mapped central region of VPg proteins from 40 different potyviruses. YXXXXLΦ above the columns denotes the location of the putative 4E-binding motif based on the motif YTDIRLI of PVA VPg. Furthermore, a putative non-canonical motif, which is similar to the motif found in eIF4G, is shown. VPg amino acid sequences aligned using ClustalW2 [61]. The color of the residue boxes is according to the Clustal X Colour Scheme. Quality is a measure of the likelihood of mutations. A high alignment quality score for a column indicates that there are no mutations, or that most of the observed mutations are favorable.

The interaction between PVA VPg and HCpro was originally found with the LexA Y2H [63] and was also detected with the GAL4 Y2H in this study (Figure 3A). All the truncated forms of VPg and HCpro (Figure S1A) were expressed in yeast (Figures S1B and S2), and our Y2H analysis indicated that only the truncated forms of VPg that contained residues 1–127, 61–127, 45–135, or 1–60 and 80–189, i.e., the central region of VPg, were able to interact with HCpro (Figure S1A). In contrast, most of the HCpro truncation mutants interacted with VPg. Only the truncated form HCpro aa1–168, which corresponds to the N-proximal part of HCpro, was unable to interact with VPg (Figure S1A). Efficient interactions with VPg, as assessed by yeast growth, were observed with HCpro mutants that contained the entire C-proximal region (residues 230–458); however, mutants lacking most of the C terminus were also able to interact with VPg (Figure S1A). These results indicated that different regions of HCpro confer interactions with VPg (central region) and the translation initiation factors (canonical 4E-binding motif; residues 345–351 in the C-proximal part) (Figure S1A) [25].

### 3.3. Interaction with VPg and 4Es in the Nucleus and Nucleolus

**Figure 4:**
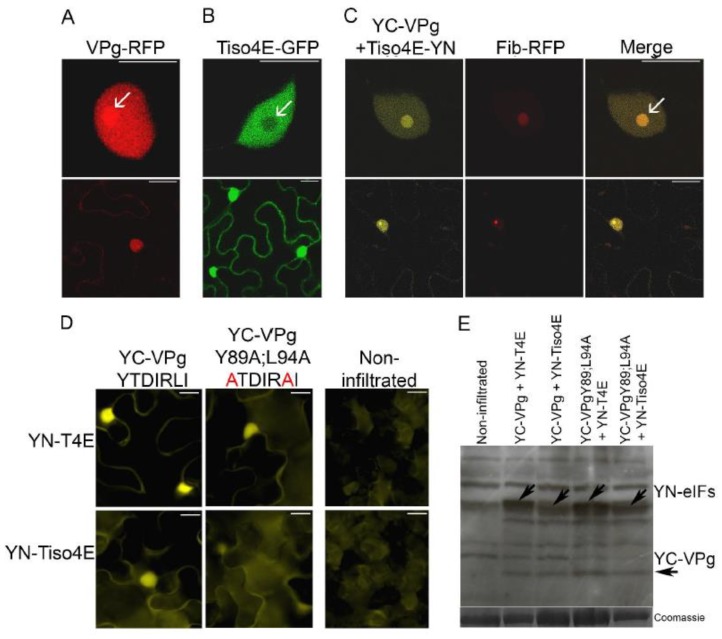
Subcellular localization of the VPg–Tiso4E interaction and the effect of amino acid substitutions in the eIF4E-binding motif of VPg on this interaction. (**A**) PVA VPg in the nucleus and nucleolus. (**B**) Tiso4E in the nucleus but not the nucleolus. (**C**) The VPg–Tiso4E interaction in the nucleus and nucleolus and its co-localization with nuclear protein fibrillarin in epidermal cells of *N. benthamiana*. Upper panels show higher-magnification images of the nucleus, and the lower panels show the entire cell. Arrows indicate the nucleolus. (**D**) VPg were fused to YC, and tobacco translation initiation factors were fused to YN. BiFC was analyzed by epifluorescence microscopy at 2 dpif. Amino acid substitutions (Y89A;L94A) within the putative canonical eIF4E-binding motif of VPg are indicated in red. (**E**) Expressed recombinant proteins were detected by western blot analysis using green fluorescent protein (GFP)-specific antibodies. The Coomassie blue-stained gel was used as the protein loading control. Correct protein sizes are indicated by arrows. Scale bars represent 10 µm.

VPg-RFP was expressed in epidermal cells of leaves of *N. benthamiana* by agroinfiltration and found to be concentrated in the nucleus and nucleolus, as observed with confocal microscopy 2 dpif (Figure 4A), consistent with previous results [36]. In similar experiments, translation initiation factor eIF(iso)4E of *N. tabacum* (tobacco) fused with GFP (Tiso4E-GFP) was detected in both the cytoplasm and nucleus, but there was no apparent signal in the nucleolus (Figure 4B).

BiFC was used to visualize protein interactions in living cells [53]. VPg was expressed by agroinfiltration in leaves of *N. benthamiana* with the C-proximal part of the YFP fused to the N terminus of VPg (YC-VPg), and Tiso4E was co-expressed with YC-VPg with the N-proximal part of YFP fused to the C terminus of Tiso4E (Tiso4E-YN). Faint YFP fluorescence resulting from the VPg–Tiso4E interaction was found in the cytoplasm, but strong signals of BiFC were observed in the nucleus and nucleolus (Figure 4C). These signals co-localized with the signals of the RFP-fused nucleolar protein fibrillarin (Fib2-RFP), which confirmed the subcellular localization of the VPg–Tiso4E interaction in the nucleus and nucleolus (Figure 4C).

### 3.4. PVA VPg Contains a 4E-Binding Motif

**Figure 5:**
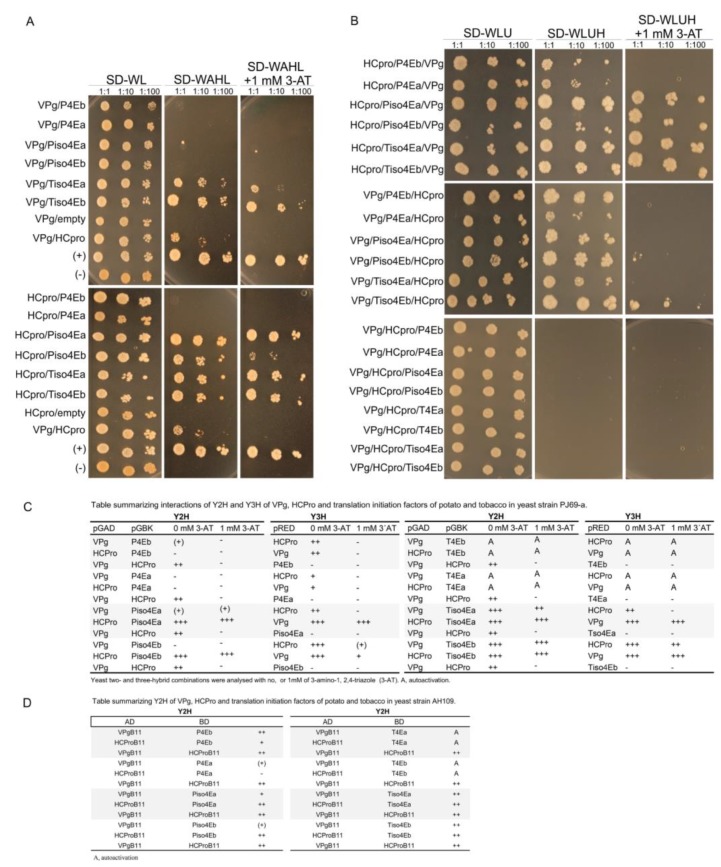
Mutual interactions between VPg, HCpro, and 4Es were tested using Y2H and Y3H in *S. cerevisiae* cells (strain PJ69-4A). Yeast cultures were grown to OD600 = 1 and then diluted 10- and 100-fold before being applied (2 µL) onto selective medium plates. Yeast growth at 30 °C was observed for up to 10 days. (**A**) Y2H results based on yeast growth. Positive transformants were selected for protein interactions by growth on synthetic defined (SD) medium lacking adenine (A), histidine (H), leucine (L), and tryptophan (W) as indicated and in the absence or presence of 1 mM 3-amino-1,2,4-triazole (3-AT). (**B**) Y3H results based on yeast growth. Positive transformants were selected for protein interactions by growth on medium lacking uracil (U), histidine (H), leucine (L), and tryptophan (W) supplemented with 1 mM 3-AT. (**C**) Table summarizing results from A and B. (**D**) Summary of Y2H interactions between VPg and HCpro with 4Es tested in *S. cerevisiae* cells (strain AH109). +++, very strong yeast growth; ++, efficient yeast growth similar to the GAL4 positive control; +, moderate yeast growth; (+), weak but detectable yeast growth; −, no detectable growth; A, auto-activation.

Interactions between PVA VPg and eIF4E and eIF(iso)4E of tobacco and potato (*Solanum tuberosum* L.) were tested using Y2H as described [25]. Four different eIF(iso)4E proteins (Tiso4Ea, Tiso4Eb, Piso4ea, and Piso4Eb) and two different eIF4E proteins (P4Ea and P4Eb) were expressed in yeast as fusion proteins with the BD of the Y2H, and all of them interacted with PVA VPg fused with the AD. However, the interactions with P4Ea and Piso4Eb were weak, as judged by the weak growth of the yeast (Figure 3A). This was expected because previous studies have shown that variants of eIF4E and eIF(iso)4E differ in their ability to interact with VPg [9]. When fused to the BD, T4Ea and T4Eb autoactivated the reporter genes and were excluded (Figure 5C,D). When VPg was fused to the BD, it showed no interaction with any translation initiation factor that was fused to the AD.

Truncated forms of VPg were prepared for Y2H analysis to determine the regions of VPg responsible for interaction with Tiso4Eb and P4Eb, both of which showed strong interactions with full-length VPg, as judged by rapid growth of the yeast. Expression of the recombinant proteins in yeast was detected by western blotting (Figure S2). Each protein interacted with at least one of the two translation initiation factors (Figure 3A). However, differences in the growth of yeast suggested that the central region of VPg (residues 61–127) was particularly important for the interaction (Figure 3B).

Our previous study [25] revealed that the HCpro sequences of 41 potyviruses available in the Descriptions of Plant Viruses database (www.dpvweb.net) contain the consensus 4E-binding motif YXXXXLΦ [17]. Here, we analyzed the VPg amino acid sequences of 40 potyviruses available in the aforementioned database and found that the central region of the PVA VPg contains a putative 4E-binding motif (YTDIRLI, residues 89–95; Figure 3C). Furthermore, a putative eIF4E-binding motif was detected in the VPg of sweet potato feathery mottle virus (SPFMV) (motif YTDILLV) and yam mosaic virus (YMV) (motif YFDMSLV) (Figure 3C). Comparison of VPg sequences among nine isolates of PVA and 36 isolates of SPFMV in GenBank revealed that the putative 4E-binding motif is conserved in all isolates (Figure S3).

The putative 4E-binding motif in PVA VPg was mutated by replacing the conserved residues Y89 and L94 with alanine (mutant VPgY89A;L94A), because the corresponding mutations in the 4E-binding motif of the HCpro of PVA abolish the interaction between HCpro and Tiso4E [25]. The BiFC assessment of the interactions between the mutated VPg (YC-VPgY89A;L94A) and YN-T4E or YN-Tiso4E resulted in weaker YFP signals than did BiFC with the wild-type VPg (Figure 4D), despite similar expression levels of the wild-type and mutated VPg proteins (Figure 4E). The mutations in YC-VPgY89A;L94A reduced, but did not abolish, the interaction of VPg with T4E and Tiso4E, which suggested that VPg is still capable of interacting with 4E/iso4E, probably via its cap-binding site.

Expression of VPg and VPgY89A;L94A fused with RFP (constructs VPg-RFP and VPgY89A;L94A-RFP, respectively) in leaves of *N. benthamiana* by agroinfiltration indicated that the amino acid substitutions, which impaired the VPg-eIF4E interaction, did not affect the nuclear and nucleolar localization of VPg (Figure S4).

### 3.5. Mutation of the 4E-Binding Motif in VPg Impairs PVA Virulence

The amino acid substitutions Y89A and L94A were introduced into VPg in an infectious cDNA clone of wild-type PVA (wtPVA) and were inoculated biolistically into the lowest full-grown leaves of *N. benthamiana* and *N. tabacum* as described [56]. All plants inoculated with wtPVA were systematically infected at 9 days post-inoculation (dpi) (Figure S5A) as determined by DAS-ELISA using a PVA-specific monoclonal antibody. The systemically infected leaves of *N. tabacum* displayed no symptoms, whereas mosaic symptoms and severe malformation were observed in the systemically infected leaves of *N. benthamiana* (data not shown). In contrast, the leaves of plants inoculated with PVAVPgY89A;L94A were virus-negative, as determined by DAS-ELISA (Figure S5A) and no symptoms were observed. wtPVA and PVAVPgY89A;L94A were engineered to express GFP from the NIa/CP junction and were agroinoculated into *N. benthamiana*. Leaf tissue inoculated with wtPVA-GFP and the non-inoculated top leaves showed strong GFP fluorescence, indicating systemic infection at 8 dpif, whereas no fluorescence was observed in the inoculated or upper leaves after agroinoculation with PVAVPgY89A;L94A-GFP (Figure S5B). At 26 dpif, the upper leaves of plants inoculated with wtPVA-GFP were malformed and fluoresced, whereas the leaves in the plants inoculated with PVAVPgY89A;L94A-GFP were symptomless and displayed no GFP fluorescence (Figure S5C). Taken together, these results indicated that mutations in the 4E-binding motif of VPg heavily reduced virulence of PVA. Predictions of the three-dimensional structure of wtVPg and the mutated VPgY89A;L94A proteins by I-TASSER [62] resulted in models with low confidence only, and thus, possible conformational changes in the mutated proteins remained obscure.

### 3.6. RNAi Suppression by VPg and HCpro May Involve 4E

**Figure 6:**
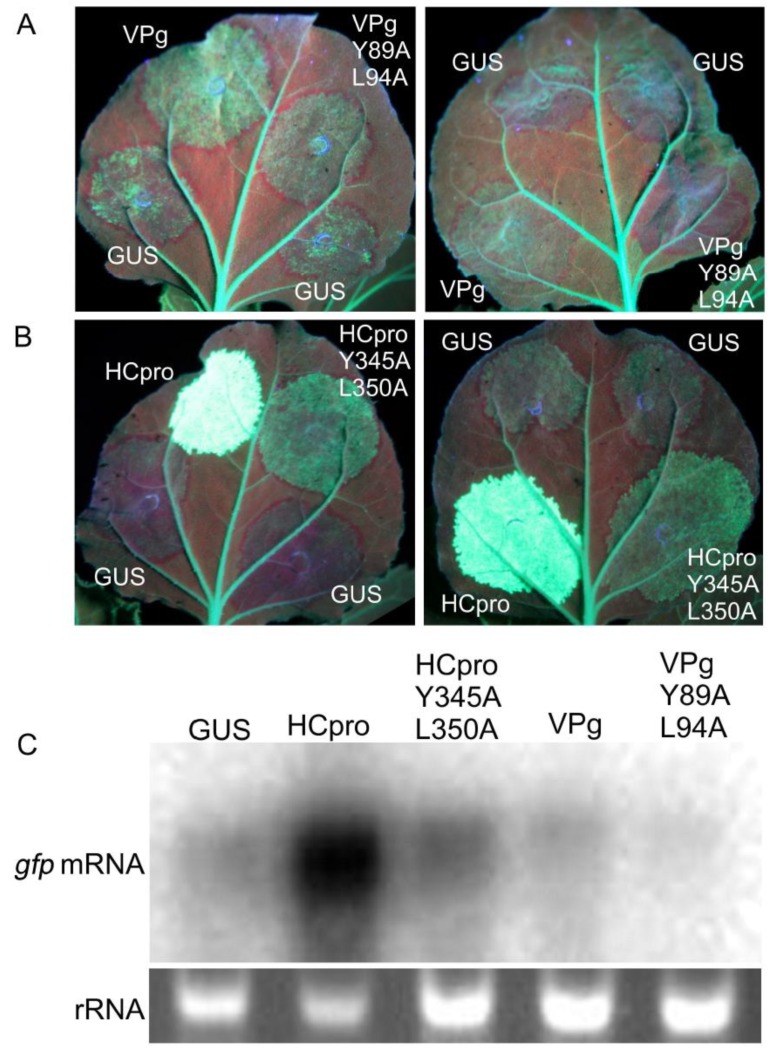
Influence of the amino acid substitutions Y89A;L94A of VPg and Y345A;L350A of HCpro on suppression of gfp silencing in leaves of transgenic line 16c of *N. benthamiana*, which constitutively expresses GFP. (**A**) Mutations Y89A;L94A in the VPg 4E-binding motif and (**B**) mutations Y345A;L350A in the HCpro 4E-binding motif reduce suppression of sense-mediated gene-silencing observed at 6 dpif and 14 dpif, respectively. Representative images are shown from two separate experiments for each construct, which differed with respect to the infiltration location (either the basal region or tip of the leaf). Green fluorescence indicates GFP expression, whereas the red color is caused by chlorophyll autofluorescence (length of leaves 6-8 cm). (**C**) Northern blot analysis of *gfp* mRNA accumulation to determine the effect of amino acid substitutions from leaf tissues collected at 6 dpif (VPg constructs) or 14 dpif (HCpro constructs and GUS). A probe corresponding to the entire *gfp* gene was used to detect *gfp* mRNA. Ethidium bromide staining of gels was used to control for equal loading.

RNAi acts as an antiviral defense mechanism and is suppressed by HCpro and VPg of PVA [36,64]. The poor virulence of PVAVPgY89A;L94A and PVAHCproY345A;L350A [25] could be associated with impaired suppression of RNAi caused by the mutations in VPg and HCpro. Sense RNA–mediated silencing of GFP expression in the full-grown leaves of *gfp*-transgenic plants of *N. benthamiana* (line 16c) [30] was achieved by overexpressing *gfp* by agroinfiltration of leaves. Overexpression initially increased fluorescence in the infiltrated leaf tissue up to 3 dpif. Subsequently, *gfp* silencing resulted in gradual disappearance of fluorescence in leaf tissues co-infiltrated to express β-glucuronidase (GUS, negative control) and VPgY89A;L94A or PVAHCproY345A;L350A. In contrast, leaf tissues infiltrated to co-express GFP and VPg or HCpro continued to fluoresce (Figure 6A,B). At 6 dpif, the leaf tissue co-infiltrated to express GFP and VPgY89A;L94A had lost all GFP fluorescence (except the fluorescence in the main veins attributed to expression of the *gfp* transgene in line 16c), whereas faint GFP fluorescence was observed in the tissues co-expressing GFP and VPg (Figure 6A). Strong GFP fluorescence in tissues co-expressing GFP and HCpro could be observed for more than 14 dpif (Figure 6B. Northern blot analysis indicated that the levels of *gpf* mRNA were higher in tissues overexpressing VPg and HCpro as compared to VPgY89A;L94A and PVAHCproY345A;L350A, respectively (Figure 6C). These results indicated that the mutations introduced into the eIF4E-binding motif in VPg and HCpro diminished the ability of VPg and HCpro to interfere with *gfp* silencing.

Subsequently, co-suppression of the *gfp* transgene in the transgenic *N. benthamiana* line 16c [30] and of ectopically expressed *gfp* was used to investigate whether Tiso4E could inhibit or enhance the interference of HCpro with gene co-suppression. Stronger GFP fluorescence was observed in leaf tissues co-infiltrated with GFP, Tiso4E, and HCpro than in leaf tissues over-expressing GFP and HCpro but not Tiso4E (Supplementary Figure S6). Similar results were consistently observed in over 20 leaves in five independent experiments. However, northern blot analysis using a radiolabeled probe specific to *gfp* showed that accumulation of *gfp* mRNA and thus the extent of siRNA remained similar no matter whether Tiso4E was included in the suppression assay.

### 3.7. Cap-Binding and eIF4G-Binding Sites of 4E: Roles in Interaction with VPg and HCpro

The amino acid residues of T4Ea implicated in binding of eIF4G (E83D, W86R) or the cap (W115A, E116A) were predicted by comparison with the previously studied eIF4Es of lettuce [15] and pea [16], respectively, and were substituted in YN-T4Ea (mutated proteins YN-T4E_E83D;W86R and YN-T4E_W115A;E116A, respectively). In a third mutant, the four substitutions were combined (YN-T4E_E83D;W86R;W115A;E116A). I-TASSER [62] was used to predict the three-dimensional structure of wtT4Ea and the three mutated T4Ea proteins. Comparison and superimposition of the models revealed no apparent structural differences (Figure S7).

**Figure 7:**
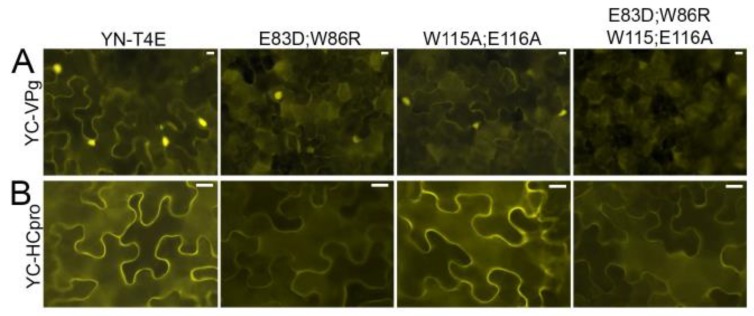
The effect of amino acid substitutions in 4G-binding residues E83D;W86R and in cap-binding residues W115A;E116A of T4Ea on its interaction with VPg and HCpro, respectively. VPg and HCpro were fused to YC, and T4Ea and T4E mutants were fused to YN. (**A**) Interactions of YC-VPg with YN-T4E and YN-T4E mutants were analyzed using BiFC at 2 dpif with an epifluorescence microscope using the same exposure time. (**B**) Interactions of YC-HCpro with YN-T4E and YN-T4E mutants were analyzed using BiFC at 3 dpif with an epifluorescence microscope using the same exposure time. Scale bars represent 10 µm.

Interaction of YC-VPg with YN-T4Ea, YN-T4E_E83D;W86R, or YN-T4E_W115A;E116A was detected by BiFC. The signals showed a similar subcellular localization in the cytoplasm and nucleus but were weaker with the mutated T4Ea proteins than with T4Ea (Figure 7A). However, when the mutant in which the four mutations were combined (YN-T4E_E83D;W86R;W115A;E116A) was co-expressed with YC-VPg, no signal was observed (Figure 7A). T4Ea and the mutated forms were expressed consistently in the infiltrated tissues (Figure S8A). These results indicated that the cap-binding domain and the eIF4G-binding site of T4E play a role in the interaction between T4Ea and VPg.

BiFC showed that YC-HCpro interacted with YN-T4E and YN-T4E_W115A;E116A, resulting in similarly strong fluorescence in both experiments, whereas much weaker fluorescence was observed following co-expression of YC-HCpro with YN-T4E_E83D;W86R or YN-T4E_E83D;W86R;W115A;E116A (Figure 7B). All mutated forms of T4Ea were expressed in the infiltrated tissues (Figure S8B). For comparison, interactions were tested using mutant HCpro in which the conserved residues of the eIF4E-binding motif had been mutated (Y345A and L350A) [25]. Co-expression of the mutated HCpro with T4Ea or Tiso4Eb resulted in much weaker fluorescence than co-expression of wtHCpro with T4Ea or Tiso4Eb (Figure S9A). All recombinant proteins were detected by western blotting (Figure S9B). Taken together, these results indicated that HCpro interacts with T4E via the eIF4G-binding domain.

Predictions of the three-dimensional structure of HCpro and the mutated HCproY345A;L350A proteins were carried out using I-TASSER [62]. Comparison and structural alignment revealed that the two models were not exactly identical (Figure S10). The motif in both models forms a helix that is extended due to the amino acid substitution Y345A in the model of HCproY345A;L350A (Figure S10). This structural change within the motif of HCpro may affect the binding to 4E and the virulence of PVA.

### 3.8. Mutual Interactions between VPg, HCpro, and 4E

A yeast three-hybrid system (Y3H) was used to study possible ternary complexes formed by VPg and HCpro with the potato and tobacco 4Es in *S. cerevisiae* cells (strain PJ69-4A) [51]. The Y3H reconstitutes the transcription of reporter genes that results from interacting proteins in a way similar to the Y2H, but the Y3H also monitors the effect of a third protein on the studied interaction. Expressed recombinant proteins in yeast were detected by western blotting (Figure S11). Interactions were detected only when VPg was fused to the AD and HCpro and the 4Es were fused to the BD.

Y2H interactions of VPg and HCpro with 4Es tested in *S. cerevisiae* cells of strain AH109 (Figure 3A; [25]) were repeated for the Y3H assay in *S. cerevisiae* cells of strain PJ69-4A (Supplementary Figure S5A,B). A similar pattern of interactions was detected in both yeast strains, except that interactions in strain PJ69-4A were somewhat weaker than in strain AH109 (Figure S1A; Figure 5A,C). Results showed that the addition of any 4E from potato or tobacco abolished the VPg–HCpro interaction (Figure 5B), suggesting that the interaction of 4E with VPg or HCpro interfered with the VPg–HCpro interaction. In contrast, the interaction between 4E and HCpro was unaffected when VPg was added as the third protein, and addition of HCpro as the third protein did not affect the VPg-4E interaction (Figure 5A,B). Addition of VPg as the third protein in Y3H assays containing HCpro and P4Ea or P4Eb, which do not interact, activated reporter gene expression, suggesting that these three proteins can form a ternary complex (Figure 5A,B). Furthermore, the weak interaction or absence of an interaction between VPg and P4Es was strengthened by the addition of HCpro in the Y3H (Figure 5A,B).

## 4. Discussion

The VPg-HCpro interaction has been detected previously in vitro and by Y2H [38,39,63]. However, it has remained unknown where these interactions between VPg and HCpro occur in potyvirus-infected plant cells. In PVA-infected plants we found that the VPg-HCpro interaction occurred in punctate bodies in the cytoplasm and in the nucleus and nucleolus. Although HCpro is intermittently localized to the nucleus [65,66,67], it is found mainly in the cytoplasm [25,65,68,69]. This study shows for the first time that HCpro can be targeted also to the nucleolus. VPg contains a bipartite nuclear localization signal that targets the majority of VPg and its precursor protein, NIa, to the nucleus and nucleolus in virus-infected cells, which is essential for viral infection [33,36]. HCpro is, therefore, likely directed to the nucleus and nucleolus via the VPg-HCpro interaction. Their interaction in the nucleus and nucleolus reported here suggests novel coordinated functions in a location where host gene expression is regulated. The interaction may also provide a way to transport HCpro to the nucleus to complete some still unknown functions during certain stages of virus infection.

Both VPg and HCpro interact with 4E [8,25]. The HCpro interaction with 4Es is mediated via the conserved, canonical 4E-binding motif, which resides between the central and C-terminal domains of HCpro (aa345–351; [25]). In 4E, the HCpro interaction site is equivalent to the eIF4G-binding domain, which was further confirmed in this study; the substitutions in the eIF4G-binding residues of 4E abolished the HCpro interaction. The data thus suggest that HCpro and eIF4G compete for 4E binding.

In contrast to the HCpro–4E interaction, which is found mainly in conjunction with 6K2-induced viral replication/translation vesicles [25], the punctate cytoplasmic bodies associated with the VPg–HCpro interaction did not overlap with the 6K2-induced vesicles. These interaction bodies are likely also not the previously described PVA-induced granules, which contain HCpro, acidic ribosomal protein P0, 4E, AGO1, oligouridylate-binding protein 1 (UBP1), and varicose, because VPg co-expression appears to block PVA-induced granule formation [70]. Instead, VPg promotes translation of PVA RNA [71,72], and HCpro can, together with VPg, synergistically increase this process [70]. Thus, VPg–HCpro interaction bodies could be involved in translation. In addition, HCpro and VPg can be found at one end of the virus particles [73]. Together, the data suggest that HCpro is involved in divergent functions in the cytoplasm during viral infection.

The VPg interaction with 4Es appears more complex than the HCpro–4E interaction. When VPg was coexpressed with 4E, the VPg–4E interaction was observed in the nucleus and nucleolus in addition to the cytoplasm. NIa (the precursor of VPg) in turnip mosaic virus also interacts with 4E in the nucleus and nucleolus for unknown reasons [74]. Previous studies demonstrate that 4E is involved in the nuclear export of selected mRNAs [75,76]. In addition, very recent data indicate that VPg can impair the nuclear export of 4E-dependent RNAs via binding to the cap-binding site and preventing RNA association with 4E in human cells [11]. Thus, the VPg–4E interaction might be the means by which potyviruses interfere with the nucleocytoplasmic trafficking of these host mRNAs to disrupt expression of some antiviral proteins. For example, different animal-infecting picornaviruses control nucleocytoplasmic trafficking by targeting nuclear pore proteins and transport factors [77]. The recently found plant protein CBE1 with a 4E-binding motif can control the expression of nuclear-encoded genes and was proposed to be the first characterized translation factor associated with plant-specific cell cycle regulators [22]. As both PVA VPg and HCpro contain a functional 4E-binding motif, they may compete with this plant factor to affect gene regulation.

Two regions of 4E have been mapped to be involved in the interaction with potyvirus VPg, one near the cap-binding domain of 4E and another in the lateral surface of 4E [16,78]. Our results indicated that PVA VPg (and maybe also SPFMV and YMV) can interact with 4E via two different domains of 4E: the cap-binding domain and the eIF4G-binding domain. Natural recessive resistance genes against different potyviruses encode 4E variants with amino acid substitutions near the cap-binding domain of 4E [8,14,79,80,81,82]. Although the cap-binding domain of 4E overlaps with the site of the VPg interaction, disrupted cap binding does not correlate with potyvirus resistance [11,15,16,79]. Instead, recent data indicate that VPg can compete with 4E for capped RNAs to inhibit host translation [11]. Plant eIF4Gs bind to 4E via their canonical 4E-binding motif but also via a second non-canonical motif consisting of hydrophobic residues, which connect to a dorsal and a lateral hydrophobic surface of 4E, respectively [18]. The non-canonical motif of eIF4G strengthens the 4E-eIF4G interaction [18]. Mutations in the lateral domain of 4E can compromise potyvirus infection and are involved in the VPg interaction [16]. Therefore, hydrophobic residues in the central part of potyviral VPg have been speculated to correspond to a non-canonical motif [18]. In this study, we show that PVA VPg uses the canonical 4E-binding motif, instead of the non-canonical motif for 4E binding. Together, the data suggest that different potyviral VPg proteins may have evolved to interact with 4E proteins via the cap-binding motif but possibly also via the canonical or non-canonical motif of eIF4G binding, both of which can potentially interfere with 4E-4G interactions. Binding to two different sites in 4E may reflect different and yet unknown 4E-mediated functions regulated by VPg during potyvirus infection.

Both our data and those from previous studies indicate that the central domain of VPg is required for the 4E interaction [8,11,83,84,85]. Furthermore, our study indicates that PVA VPg contains a functional 4E-binding motif in the central part of VPg (aa89–95). This motif likely mediates VPg binding to 4E via the canonical eIF4G-binding domain. Other residues in the central region of PVA VPg are, however, also involved in 4E binding and may mediate the binding to the cap-binding domain of 4E. The data are, thus, similar to findings from other studies that have mapped the central part of VPg being important for 4E binding. Structural studies on PVY VPg bound to human eIF4E and plant eIFiso4E indicate that the region aa103–121 is important for the interaction, with aa108–119 being most significant [11]. Leonard et al. [83] narrowed down the iso4E (from *A. thaliana*) interaction site of turnip mosaic virus VPg to aa59–93 with aspartic acid 77 being especially important. Interestingly, this region overlaps with the speculated non-canonical motif of eIF4G binding in VPg (aa70–80 in PVA). Furthermore, mutations in the central region of VPg overcome 4E-mediated resistance in several potyvirus-host systems [e.g., 8,84,85].

**Figure 8:**
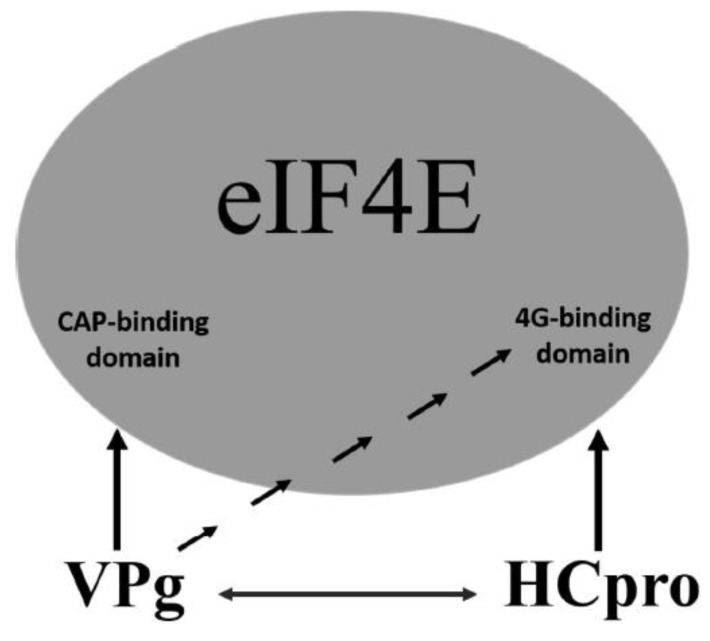
Schematic model of the two distinct binding sites of translation factor eIF4E: one binding site for the cap and another binding site for eIF4G. PVA VPg has two binding domains for eIF4E/eIF(iso)4E. One is capable of binding in close proximity to the cap-binding pocket of eIF4E, and another can bind to the eIF4G-binding domain. The YXXXXLΦ-binding domain (eIF4G-binding site) is located about 180 degrees from the predicted cap-binding pocket [16,78]. Considering that HCpro can also bind to the eIF4G-binding site by a conserved eIF4E-binding motif, it is possible that potyviruses have the capacity to control various eIF4E functions in addition to their anticipated effects on translation initiation. Arrows indicate interactions between proteins or protein domains.

An additional level of complexity in potyvirus-4E interactions is introduced by the mutual interactions of VPg with HCpro. Although in PVA VPg, the regions required for interaction with HCpro and 4E overlap in the central part of VPg, distinct regions in HCpro confer interactions with VPg and 4E. Furthermore, our data indicated that interactions of VPg and HCpro with 4E dominate over the mutual interaction of VPg and HCpro. These results suggest a model in which VPg and HCpro bind 4E primarily via the cap-binding domain and the eIF4G-binding domain of 4E, respectively, and VPg–HCpro interactions may act as a regulatory mechanism (Figure 8). VPg may be able to control the HCpro–4E interaction by binding to the eIF4G-binding domain of 4E. In contrast, HCpro may be able to control interactions of VPg with 4E, as HCpro interacts with the central region of VPg, which also contains the binding sites for 4E. In addition to 4E, HCpro also interacts with many other host proteins, mostly for unknown reasons [25,54,86]; and references therein], whereas VPg as an intrinsic disordered protein [87] can bind different proteins [88].

Both HCpro and VPg suppress antiviral RNAi [29,30,31,36]. In this study, mutations introduced into the 4E-binding motif in VPg and HCpro reduced levels of *gfp* mRNA and diminished GFP fluorescence, suggesting that the interactions of VPg and HCpro with 4E may contribute to RNAi suppression. Consistent with this, stronger GFP fluorescence was observed in leaf tissues co-infiltrated with GFP, Tiso4E, and HCpro as compared with leaf tissues over-expressing GFP and HCpro but not Tiso4E. The accumulation of *gfp* mRNA and siRNA remained, however, similar no matter whether Tiso4E was absent or present, suggesting that the strong GFP fluorescence could be associated with enhanced translation caused by overexpressed Tiso4E rather than enhanced co-suppression. Hafren et al. [70] have also observed that amino acid substitutions in the 4E-binding motif of HCpro reduce silencing suppressor activity of HCpro. However, the same mutations in the 4E-binding motif of HCpro also affect other HCpro functions, i.e., the mutations reduce the formation of PVA-induced cytoplasmic granules and reduce viral RNA accumulation resulting from VPg and HCpro co-expression [70]. The functions of the RNA-silencing and RNA decay pathways partly overlap and may co-operate with respect to virus defense in plants based on recent data [32]. Both VPg and HCpro bind essential components of the RNA decay system, decapping protein 2 (DCL2) and exoribonuclease 4 (XRN4), respectively, and suppress RNA decay in *N. benthamiana* [32]. The VPg interaction targets DCL2 to the nucleus and, therefore, prevents the accumulation of cytoplasmic DCL1/DCL2 RNA decay complexes, whereas HCpro inhibits slicing activity of XRN4 [32]. The VPg-4E interaction also likely provides stability for virus RNA. Thus, mutations in 4E-binding motifs of VPg and HCpro may also be associated with reduced suppression of RNA decay.

It is well established that potyviruses have evolved to mediate host interactions via the 4E translation initiation factors. Furthermore, it is known that mutations in the cap-binding domain of 4E in the host plant, or mutations in VPg of the virus, can make the host-virus interaction incompatible, which prevents normal infection [10]. The results presented here suggest, however, that the VPg–4E interaction is only one component, albeit an important one, of the interaction network between potyviral proteins and 4Es. Our findings suggest that potyviruses control and utilize the host translation initiation factors in a sophisticated way for functions that, in general, are poorly understood. New dimensions of the interaction network emerged following the discovery of the common occurrence of the 4E-binding motif in HCpro [25], and the new results reported here reveal that a few potyviruses, such as PVA, SPFMV, and YMV, also have the 4E-binding motif in their VPg proteins. In addition, some other potyviruses may use the non-canonical motif for 4G binding for the VPg–4E interaction. Whereas the 4E-binding motif exists in cellular proteins that regulate translation in eukaryotes [17], their existence has only recently been found in the proteins of viruses [25,89]. It is intriguing whether 4E gene alleles containing mutations in the 4G-binding domain exist in plant populations and whether they could confer resistance to potyviruses. Such alleles seem worthwhile to search or engineer for the needs of resistance breeding, especially concerning resistance to PVA, SPFMV, and YMV, which infect important crop plants and contain the 4E-binding motif in two proteins.

## Supplementary Materials

The following are available online at https://www.mdpi.com/1999-4915/11/12/1158/s1, Figure S1: mapping of the regions in VPg and HCpro involved in the VPg–HCpro interaction, Figure S2: recombinant VPg, 4E, and HCpro proteins expressed in yeast, Figure S3: multiple sequence alignments of the region comprising amino acids 85–100 of VPg from different isolates of potato virus A (PVA) and sweet potato feathery mottle virus (SPFMV), Figure S4: expression of mRFP-tagged VPg in *N. benthamiana*, Figure S5: influence of the amino acid substitutions Y89A;L94A of VPg on PVA virulence, Figure S6: influence of Tiso4E on HCpro interference with gene co-suppression, Figure S7: three-dimensional structural models of wild-type T4E and three mutants as predicted with I-TASSER, Figure S8: expressed recombinant proteins detected in agroinfiltrated leaves in BiFC assay by western blotting using GFP antibodies, Figure S9: effect of the amino acid substitutions Y345A and L350A within the eIF4E-binding motif of HCpro on interactions with translation factors, as detected by BiFC, Figure S10: three-dimensional structural models of wild-type HCpro and the HCpro mutant with amino acid substitutions Y345 and L350 in the 4E-binding motif predicted with I-TASSER, Figure S11: proteins expressed from the Y3H-Vector pRED-NLSa were detected by western blot analysis using protein-specific antibodies, Table S1: primers for amplifying genes cloned into the activation-domain vector (pGADT7) or the DNA binding-domain vector (pGBKT7) of the Y2H, and for the cloning of genes into the Y3H vector pRED-NLSa, Table S2: primers used to construct binary vectors in this study.
