# Supplementary material for: A Novel Interaction Network Used by Potyviruses in Virus–Host Interactions at the Protein Level
Source: Viruses. 2019 Dec 14;11(12):1158. doi: 10.3390/v11121158 (PMC6950583; doi:10.3390/v11121158)
Supplement: Supplementary file 1 [file viruses-11-01158-s001.pdf]

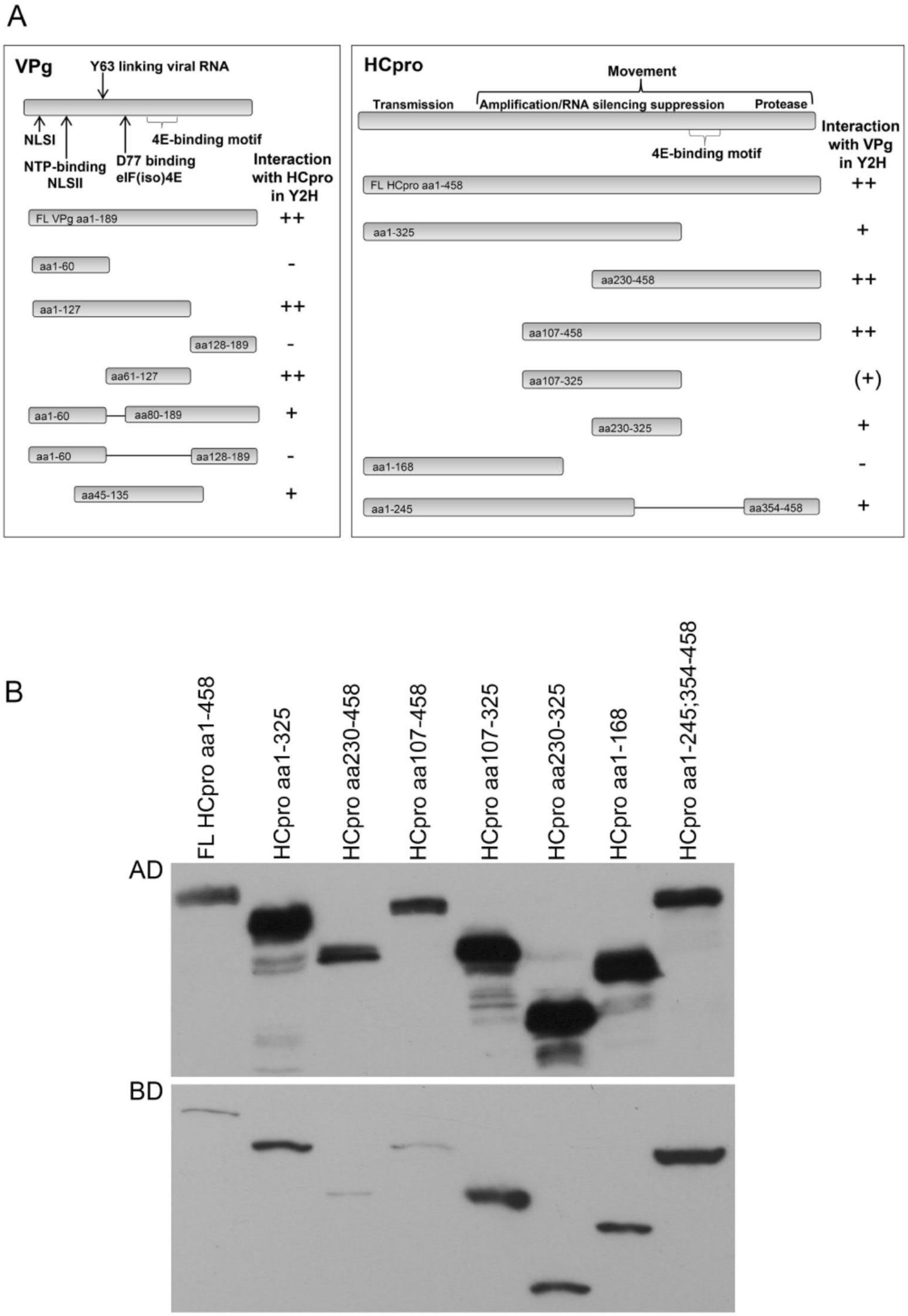

Figure S1. Mapping of the regions in VPg and HCpro involved in the VPg-HCpro interaction. (**A**) Schematic figure summarizing the detected interactions as revealed by yeast growth (*Saccharomyces cerevisiae*, strain AH109) using the Matchmaker GAL4 (Clontech) Y2H assay. 14 days after plating on selective medium: ++, efficient yeast growth similar to the GAL4 positive control; +, moderate yeast growth; (+), weak but detectable yeast growth; –, no detectable growth. The regions of the full-length (FL) and truncated forms of VPg and HCpro are indicated by numbers of the amino acid residues they are composed of. NLS, nuclear localization signal; NTP, nucleotide triphosphate. (**B**) Full-length and truncated HCpro proteins expressed in yeast and detected with antibodies specific to the activation domain (AD) or binding domain (BD) by western blot analysis. See Figure S3 for expression of full-length and truncated VPg.

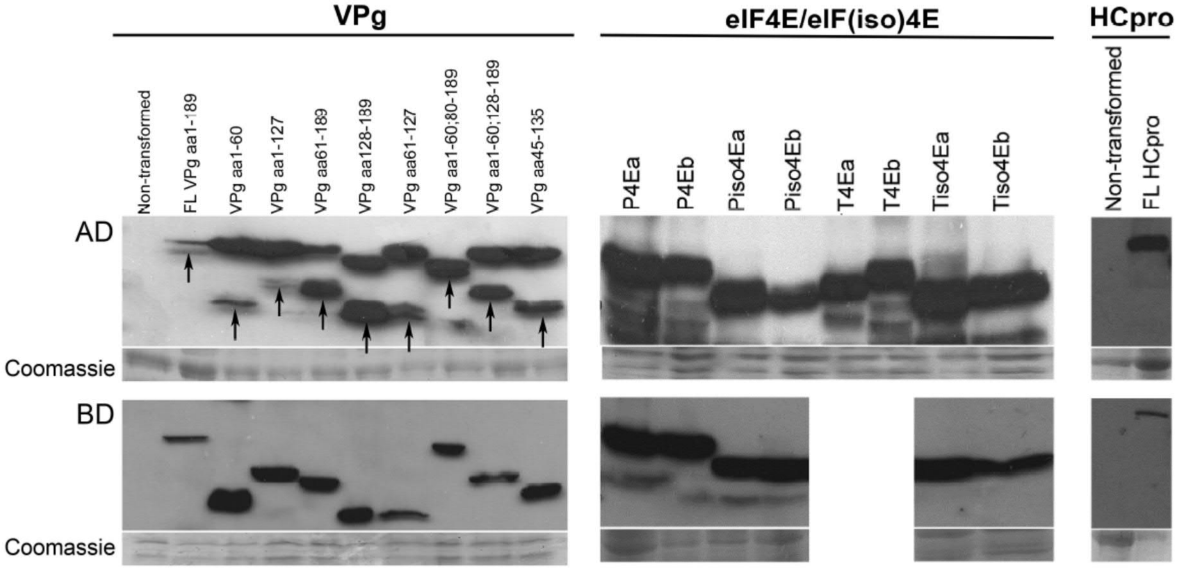

Figure S2. Recombinant VPg, 4E, and HCpro proteins expressed in yeast. Recombinant proteins from Y2H assays were detected with monoclonal antibodies specific to the activation domain (AD) or DNA-binding domain (BD) by western blot analysis. The full-length and truncated forms of VPg, the different forms (a and b) of translation initiation factors eIF4E (4E) and eIF(iso)4E (iso4E) from potato (P) and tobacco (T), and full-length HCpro were detected. A Coomassie blue–stained SDS gel served as a protein loading control. The expected sizes of fusion proteins carrying the AD domain are indicated by arrows.

A

```
AJ131400|VPg_protein|5688-6254 LDESPYTDIRLIQSQFS 100
AJ277992|VPg_protein|515-1081  LDESPYTDIRLIQSQFS 100
F543212|VPg_protein|5688-6254  LDESPYTDIRLIQSQFS 100
AJ131402|VPg_protein|5688-6254 LDESPYTDIRLIQSQFS 100
GU144321|VPg_protein|5687-6253 LDESPYTDIRLIQSQFS 100
AJ132007|VPg_protein|515-1081  LDESPYTDIRLIQSQFS 100
AJ131401|VPg_protein|5688-6254 LDESPYTDIRLIQSQFS 100
AJ296311|VPg_protein|5688-6254 LDESPYTDIRLIQSQFS 100
Z21670|VPg_protein|5688-6254   LDESPYTDIRLIQSQFS 100
                               *****************
```

B

```
AB465608|VPg_protein|6859-7434 DDSPYTDILLVQEKIG 100
FJ795779|VPg_protein|187-762   DDSPYTDILLVQEKIG 100
FJ155666|VPg_protein|6960-7535 DDSPYTDILLVQEKIG 100
AB439206|VPg_protein|6784-7359 DDSPYTDILLVQEKIG 100
FJ795764|VPg_protein|187-762   DDSPYTDILLVQEKIG 100
FJ795765|VPg_protein|187-762   DDSPYTDILLVQEKIG 100
FJ795766|VPg_protein|187-762   DDSPYTDILLVQEKIG 100
FJ795758|VPg_protein|187-762   DDSPYTDILLVQEKIG 100
FJ795767|VPg_protein|187-762   DDSPYTDILLVQEKIG 100
FJ795776|VPg_protein|187-762   DDSPYTDILLVQERIG 100
FJ795777|VPg_protein|187-762   DDSPYTDILLVQERIG 100
FJ795775|VPg_protein|187-762   DDSPYTDILLVQERIG 100
FJ795772|VPg_protein|187-762   DDSPYTDILLVQERIG 100
FJ795771|VPg_protein|187-762   DDSPYTDILLVQERIG 100
FJ795770|VPg_protein|187-762   DDSPYTDILLVQERIG 100
FJ795769|VPg_protein|187-762   DDSPYTDILLVQERIG 100
D86371|VPg_protein|6784-7359   DDSPYTDILLVQERIG 100
FJ795773|VPg_protein|187-762   DDSPYTDILLVQERIG 100
FJ795762|VPg_protein|187-762   DDSPYTDILLVQERIG 100
FJ795763|VPg_protein|187-762   DDSPYTDILLVQERIG 100
FJ795759|VPg_protein|187-762   GGPPYTDMSLFKKKIG 100
FJ795760|VPg_protein|187-762   YDSPYTNILLVQEKIG 100
FJ795761|VPg_protein|187-762   DDSPYTDILLVQEKIG 100
FJ795774|VPg_protein|187-762   DDSPYTDILLVQERIG 100
FJ795768|VPg_protein|187-762   DDSPYTDILLVQEKIG 100
FJ795778|VPg_protein|187-762   DDSPYTDILLVQEKIG 100
FJ795780|VPg_protein|187-762   DDSPYTDILLVQEKIG 100
FJ795749|VPg_protein|994-1569  DDSPYTDIQLVQEKIG 100
FJ795750|VPg_protein|994-1569  DDSPYTDIQLVQEKIG 100
FJ795753|VPg_protein|301-876   DDSPYTDIQLVQEKIG 100
FJ795754|VPg_protein|301-876   DDSPYTDIQLVQEKIG 100
FJ795756|VPg_protein|301-876   DDSPYTDIQLVQEKIG 100
FJ795751|VPg_protein|994-1569  DDSPYTDIQLVQEKIG 100
FJ795755|VPg_protein|301-876   DDSPYTDIQLVQEKIG 100
FJ795757|VPg_protein|301-876   DDSPYTDIQLVQEKIG 100
FJ795752|VPg_protein|994-1569  DDSPYTDIQLVQEKIG 100
                                ..***:: *.:::**
```

Figure S3. Multiple Sequence Alignments of the Region Comprising Amino Acids 85–100 of VPg from different isolates of potato virus A (PVA) and sweet potato feathery mottle virus (SPFMV). The eIF4E-binding motif YXXXLΦ is indicated in red. An asterisk indicates a fully conserved residue, a colon indicates strongly similar properties, and a period indicates weakly similar properties. (**A**) Alignment of PVA isolates. (**B**) Alignment of SPFMV isolates.

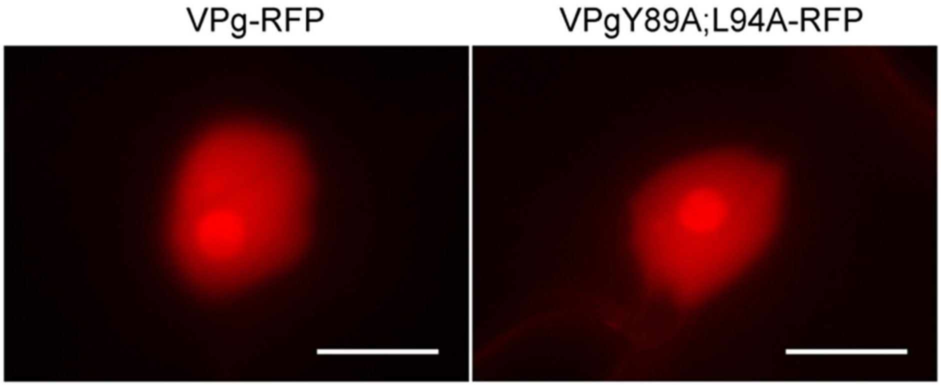

Figure S4. Expression of mRFP-tagged VPg in *N. benthamiana*. Expression of mRFP-tagged VPg and the mRFP-tagged double amino acid substitution mutant VPgL89A;Y94A revealed similar localization in the nucleus and nucleolus. Scale bars represent 10 µm.

A

Detection of PVA by DAS-ELISA

	Nicotiana benthamiana							
	9 dpi				24 dpi			
	EXP I		EXP II		EXP I		EXP II	
Construct	No.[a]	µg virus/g leaf[b]	No.[a]	µg virus/g leaf[b]	No.[a].	µg virus/g leaf[b]	No.[a]	µg virus/g leaf[b]
wtPVA	3/3	116 (5)	3/3	230 (50)	3/3	106 (11)	3/3	531 (100)
PVAVPgY89A;L94A	0/3	-	0/3	-	0/3	-	0/3	-
MOCK	0/2	-	0/2	-	0/2	-	0/2	-

	Nicotiana tabacum							
	9 dpi				24 dpi			
	EXP I		EXP II		EXP I		EXP II	
Construct	No.[a]	µg virus/g leaf[b]	No.[a].	µg virus/g leaf[b]	No.[a]	µg virus/g leaf[b]	No.[a]	µg virus/g leaf[b]
wtPVA	3/3	2 (1)	3/3	1 (0.4)	3/3	0.6 (0.5)	3/3	7 (2)
PVAVPgY89A;L94A	0/3	-	0/3	-	0/3	-	0/3	-
MOCK	0/2	-	0/2	-	0/2	-	0/2	-

[a] The number of systematically infected plants out of the number of inoculated plants.
[b] Virus amounts in upper non-inoculated leaves tested at 9 and 24 days post inoculation (dpi). Virus amounts were estimated using a standard curve of known amounts of purified PVA virions (ug virus per gram of fresh leaf; SD of mean in parenthesis).

B 8 dpif C 26 dpif

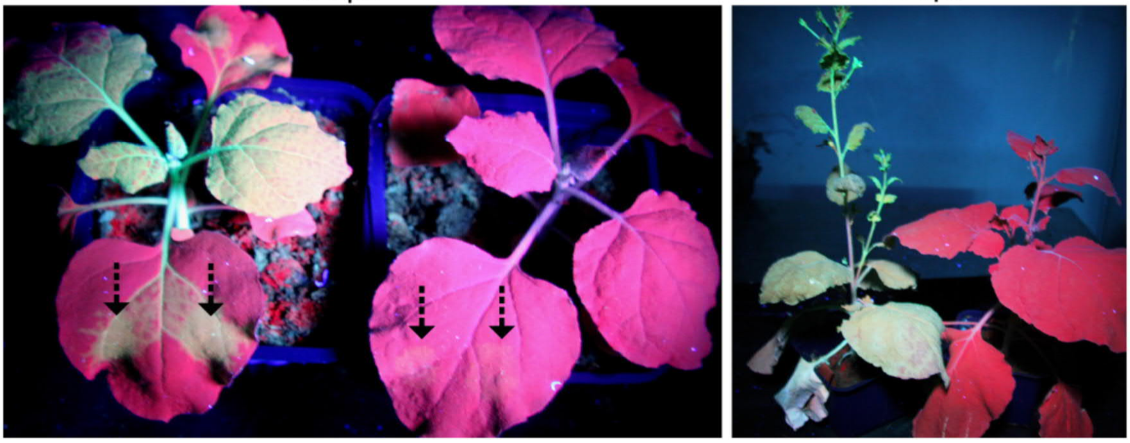

Figure S5. Influence of the amino acid substitutions Y89A;L94A of VPg on PVA virulence. **(A)** Infectious cDNA clones were inoculated biolistically into *N. benthamiana* and *N. tabacum. P*resence of PVA was detected in the upper non-inoculated leaves by DAS-ELISA 9 dpi. Two independent experiments (EXP I and II) were carried out for each construct. Mock plants were inoculated with gold particles lacking virus. -, no virus detected. (**B**) Wild-type *N. benthamiana* infiltrated with *Agrobacterium* expressing an infectious clone of wtPVA-GFP (left) and PVAVPgY89A;L94A-GFP (right) at 8 days post-infiltration (dpif). Arrows indicate infiltration area. (**C**) Wild-type *N. benthamiana* infiltrated with *Agrobacterium* expressing wtPVA-GFP (left) and PVAVPgY89A;L94A-GFP (right) at 26 dpif. Green fluorescence indicates GFP expression, whereas the red color is chlorophyll autofluorescence.

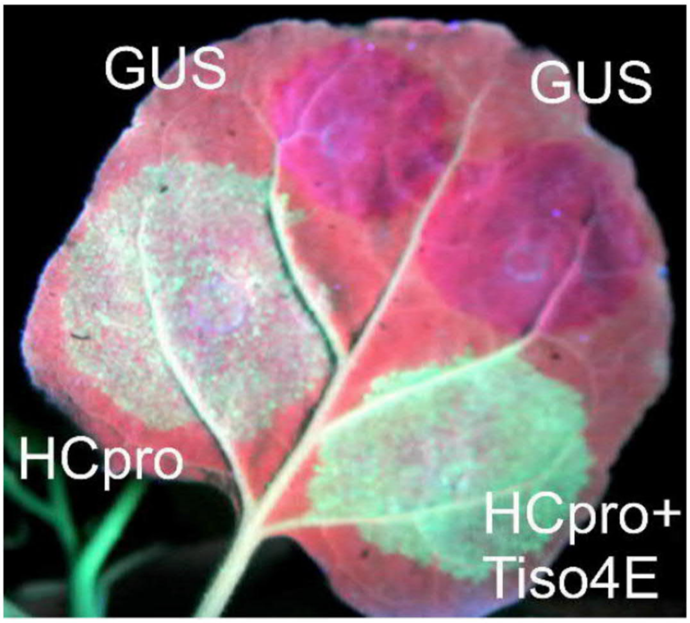

Figure S6. Influence of Tiso4E on HCpro Interference with Gene Co-Suppression. To investigate whether Tiso4E could inhibit or enhance the HCpro interference with GFP co-suppression, a co-infiltration assay was carried out**.** GFP accumulation in infiltrated leaves was detected using a UV-lamp 16 dpif. Green fluorescence indicates GFP expression, whereas the red color is chlorophyll autofluorescence.

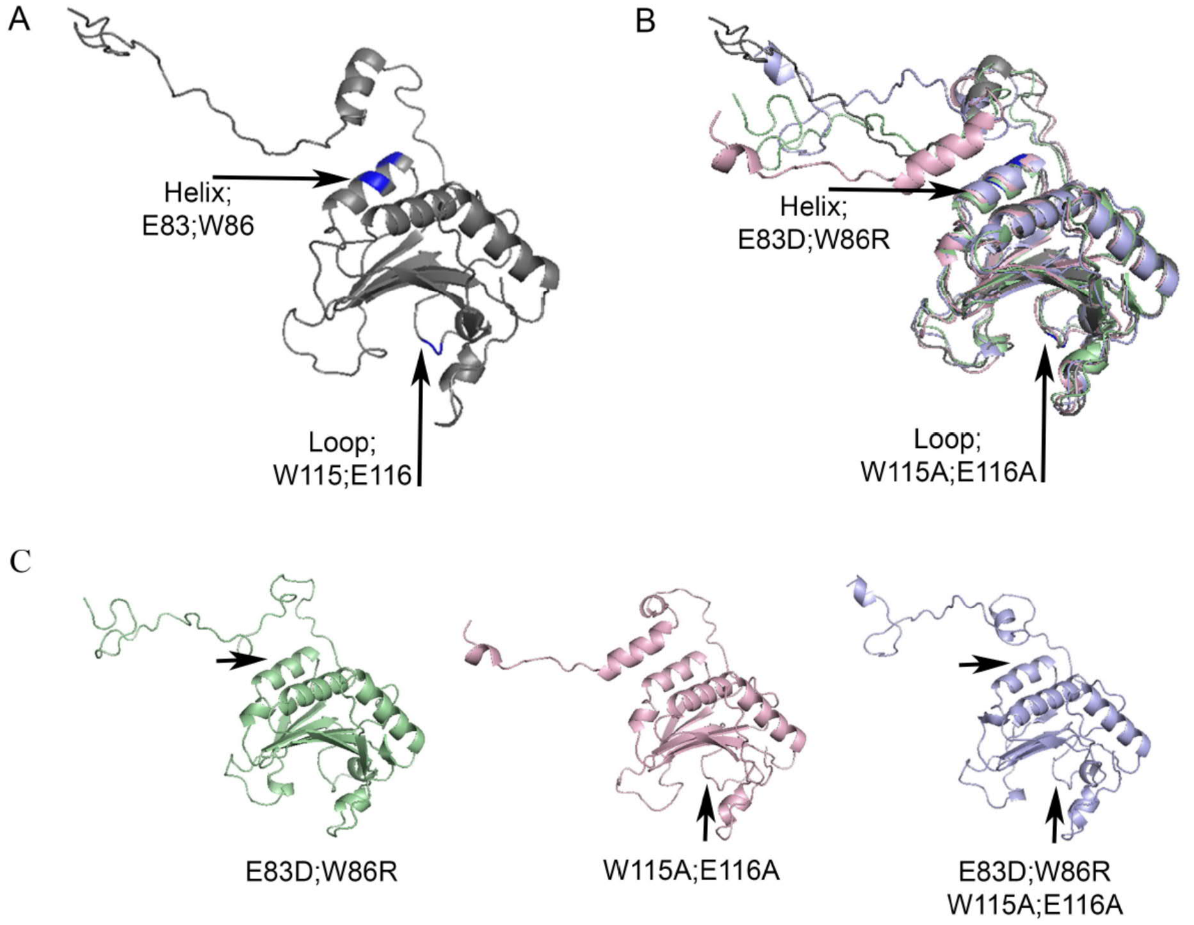

Figure S7. Three-Dimensional Structural Models of Wild-Type T4E and Three Mutants as Predicted with I-TASSER. (**A**) Amino acids involved in 4G-binding (helix) or cap-binding (loop) of T4Ea are indicated by arrows and highlighted in blue. (**B**) Superimposed wild-type T4E (gray) and T4E mutants. (**C**) The different T4E mutants with altered amino acids in the 4G-binding site (green, E83D;W86R), in the cap-binding site (pink, W115A;E116A) and in both the 4G- and the cap-binding sites (purple, E83D;W86R;W115A;E116A). Arrows in **B** and **C** indicate the locations of mutations.

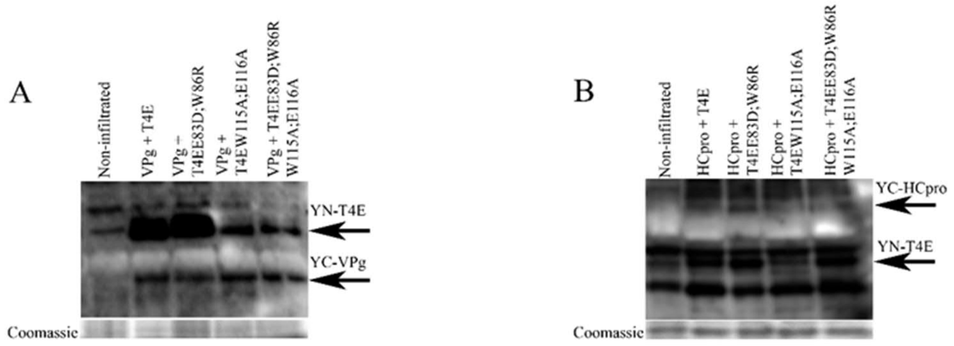

Figure S8. Expressed Recombinant Proteins Detected in Agroinfiltrated leaves in BiFC assay by Western Blotting using GFP Antibodies. (**A**) Detection of YC-VPg and translation factors T4E fused to YN. (**B**) Detection of YC-HCpro and translation factors T4E fused to YN.

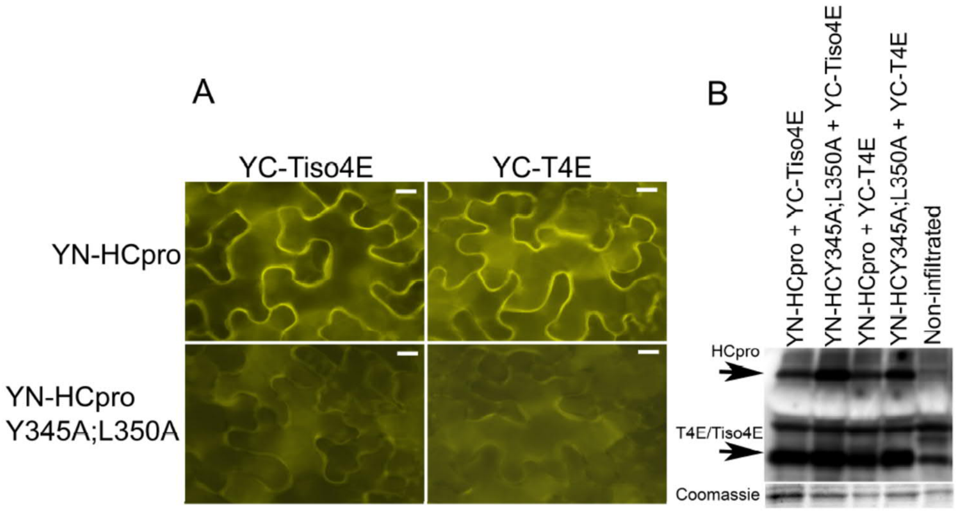

Figure S9. Effect of the Amino Acid Substitutions Y345A and L350A within the eIF4E-Binding Motif of HCpro on Interactions with Translation Factors, as detected by BiFC. (**A**) Wild-type HCpro and the HCpro mutant with amino acids Y345 and L350 in the 4E-binding motif substituted with alanines were tested for interactions with Tiso4E and T4E using BiFC. Epidermal cells of *Nicotiana benthamiana* were observed with an epifluorescence microscope 3 dpif. Scale bars represent 10 µm. (**B**) Expressed recombinant proteins were detected by western blot analysis using GFP-specific antibodies. A Coomassie blue–stained SDS gel served as a protein-loading control. Arrows indicate the positions of the YN- and YC-fused recombinant proteins.

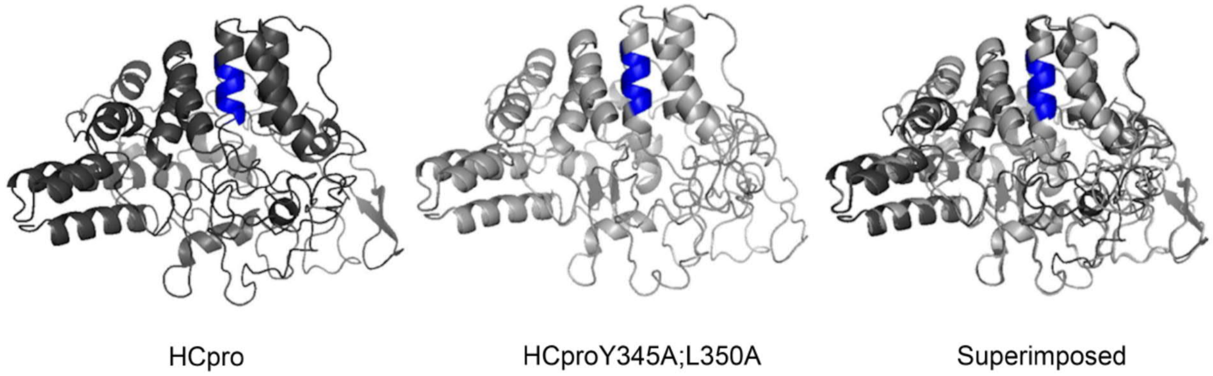

Figure S10. Three-dimensional structural models of wild-type HCpro and the HCpro mutant with amino acid substitutions Y345 and L350 in the 4E-binding motif predicted with I-TASSER. The 4E-binding motif is indicated in blue.

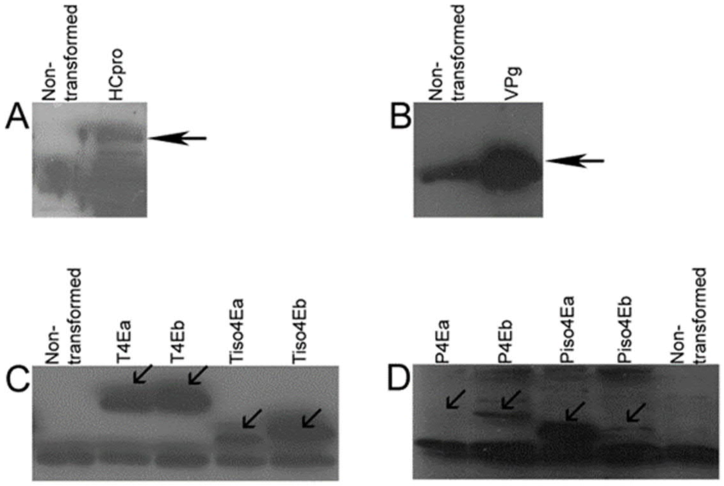

Figure S11. Proteins Expressed from the Y3H-Vector pRED-NLSa Were detected by Western Blot Analysis using Protein-Specific Antibodies. (**A**) HCpro. (**B**) VPg. (**C**) 4E translation factors of tobacco. (**D**) 4E translation factors of potato. Arrows indicate proteins of the expected size. Non-transformed yeast was used as a control.

Table S1. Primers for amplifying genes cloned into the activation-domain vector (pGADT7) or the DNA binding–domain vector (pGBKT7) of the Y2H, and for the cloning of genes into the Y3H vector pRED-NLSa.

Construct	Primer[a]	Sequence (5´ to 3´)
pGAD/pGBK VPg and<br>pGAD/pGBK VPgY89A;L94A	FwdVPgBamHI (*Bam*HI)<br>RevVPgSalI (*Sal*I)	CGGGATCCATGGCTATAATAAGCGACAG<br>CGGTCGACCTCGAATTCAACCGACTC
pGAD/pGBK VPg aa1-60	FwdVPgBamHI (*Bam*HI)<br>VPg60REVSalI (*Sal*I)	CGGGATCCATGGCTATAATAAGCGACAG<br>GCTGGTCGACCACGAACCTGTGGTTCTTTTTC
pGAD/pGBK aa1-127	FwdVPgBamHI (*Bam*HI)<br>VPg127REVSalI (*Sal*I)	CGGGATCCATGGCTATAATAAGCGACAG<br>GCTGGTCGACTAAATACCCTTGGACACCTGG
pGAD/pGBK aa 61-189	VPg61FWDBamHI (*Bam*HI)<br>RevVPgSalI (*Sal*I)	CGGGATCCAGAATATGTATGGTTTTGATCC<br>CGGTCGACCTCGAATTCAACCGACTC
pGAD/pGBK aa128-189	VPg128FWD (*Bam*HI/*Xho*I)<br>RevVPgSalI (*Sal*I)	CGGGATCCCGCTCGAGGTGAAGGATAAAACCTCAC<br>CGGTCGACCTCGAATTCAACCGACTC
pGAD/pGBK aa61-127	VPg61FWDBamHI (*Bam*HI)<br>VPg127REVSalI (*Sal*I)	CGGGATCCAGAATATGTATGGTTTTGATCC<br>GCTGGTCGACTAAATACCCTTGGACACCTGG
pGAD/pGBK aa1-60; 80-189	VPg80FWD (*Bam*HI/*Xho*I)<br>RevVPgSalI (*Sal*I)	CGGGATCCCGCTCGAGACTGGCTACACACTAGATG<br>CGGTCGACCTCGAATTCAACCGACTC
pGAD/pGBK aa1-60; 128-189		VPg aa1–60 fragment ligated to VPg aa128–189
pGAD/pGBK aa45-135	VPgaa45Fwd (*Bam*HI)<br>VPgaa135Rev (*Sal*I)	CCGGGATCCATACAAAGGGCAAAAC<br>CCGGTCGACTATTTGTGAGGTTTTATC
pREDVPg	FWDpREDVPgNruI (*Nru*I)<br>RevVPgSalI (*Sal*I)	CGTCGCGAGGCTATAATAAGCGAC<br>CGGTCGACCTCGAATTCAACCGACTC
pREDHCpro	FWDpREDHCproNruI (*Nru*I)<br>FLHCpro-Rev (*Sal*I)	CGTCGCGATATTCAACAGGGGATG<br>ATATGTCGACCTATCCAACCCTGTAGTGCTTC (Ala-Poikela et al., 2011)
pREDP4Ea and<br>pREDP4Eb	FWDpREDP4ENruI (*Nru*I)<br>P4E-YTHS-Rev (*Sal*I)	CGTCGCGAATGGCAGCAGCTGAAATG<br>CGACGTCGACCTATACGGTGTAACGATTCTTG (Ala-Poikela et al., 2011)
pREDPiso4Ea and<br>pREDPiso4Eb	FWDpREDT/Piso4ENruI (*Nru*I)<br>Piso4E-YTHS-Rev (*Sal*I)	CCTCGCGAATGGCCACCGAAGCACC<br>CGACGTCGACTCACACAGTATATCGGCTCTTAG (Ala-Poikela et al., 2011)
pREDT4Ea and<br>pREDT4Eb	FWDpREDT4ENruI (*Nru*I)<br>T4E-YTHS-Rev (*Sal*I)	CGTCGCGAATGGTTGATGAAGTAGAG<br>CGGTCGACCTACACAAGAATAACGATTC (Ala-Poikela et al., 2011)
pREDTiso4Ea and<br>pREDTiso4Eb	FWDpREDT/Piso4ENruI (*Nru*I)<br>Tiso4E-YTHS-Rev (*Xho*I)	CCTCGCGAATGGCCACCGAAGCACC<br>CCGCTCGAGCTCGACTCACACAGTATATC (Ala-Poikela et al., 2011)

[a] The restriction site for the enzyme indicated in parentheses was added to the primer sequence and is underlined.

Table S2. Primers used to construct binary vectors in this study.

Construct	Primer[a]	Sequence (5´ to 3´)[b]
pRTVPg and pRTVPgY89A;L94A	VPgFWD (*Xho*I)	CCGCTCGAGATGGCTATAATAAGCGAC
	VPgREV (*Xho*I)	CCTCTCGAGCTCGAATTCAACCGACTC
	FWD(*Bsp*HI)	CCCTCATGATGGGCTATAATAAGCGACAGAG
	VPgREV (*Bsp*HI)	CCCTCATGACTACTCGAATTCAACCGACTCTT
pBLUeVPgY89A;L94A	VPgY89A;L94A-SENSE	CTAGATGAGAGTCCC**GCT**ACTGATATCAGG**GCA**ATTCAGAGTC
	VPgY89A;L94A-ANTISENSE	GACTCTGAAT**TGC**CCTGATATCAGT**AGC**GGGACTCTCATCTAG
pRTT4EE83D;W86R	T4E E83D_W86R_SENSE	TTTACACCTTCTCCACTGTC**GAC**GATTTT**CGG**AGTGTGTACAACAATATC
	T4E E83D_W86R_ANTISENSE	GATATTGTTGTACACACT**CCG**AAAATC**GTC**GACAGTGGAGAAGGTGTAAA
pRTT4EW115A;E116A	T4E W115A;E116A_SENSE	GAATAAAATTGAGCCAAAG**GCGGCG**GATCCTGTCTGCGCCAACGGAGG
	T4EW115A;E116A_ANTISENSE	CCTCCGTTGGCGCAGACAGGATC**CGCCGC**CTTTGGCTCAATTTTATTC
pLH-RFP-HCpro	RFP-XhoI_F (*Xho*I)	ATACTCGAGATGGCCTCCTCCGAGGACG
	RFP-PciI_R (*Pci*I)	ATATACATGTTGGCGCCGGTGGAGTGGC
	HCProFWDNcoI (*Nco*I)	GCGCCATGGCGTATTCAACAGGGGATGTTTTC
	HCProREVNcoI (*Nco*I)	GCGCCATGGCTATCCAACCCTGTAGTGCTTC
pLH-VPgRFP and pLH-VPgY89A;L94A	VPgFWD (*Xho*I)	CCGCTCGAGATGGCTATAATAAGCGAC
	VPgREV (*Xho*I)	CCTCTCGAGCTCGAATTCAACCGACTC
pA-Tiso4E-GFP	Tiso4E-Fwd (*Xho*I)	CCGCTCGAGATGGCCACCGAAGCACCGG
	Tiso4E-Rev (*Nco*I)	CGGCCATGGCTACTCGACTCACACAGTATATC
pRTYN-HCproY345A;L350A	PVA-HCpro-Fwd (*Nco*I)	GCGCCATGGCGTATTCAACAGGGGATGTTTTC
	PVA-HCpro-Rev (*Nco*I)	GCGCCATGGCTATCCAACCCTGTAGTGCTTC

[a] The restriction site for the enzyme indicated in parentheses was added to the primer sequence and is underlined.

[b] Nucleotide codons encoding mutated amino acids are denoted in bold.
